# Efficacy and safety of first-line treatments for advanced hepatocellular carcinoma patients: a systematic review and network meta-analysis

**DOI:** 10.3389/fimmu.2024.1430196

**Published:** 2024-09-17

**Authors:** Jingyi Li, Bowen Yang, Zan Teng, Yunpeng Liu, Danni Li, Xiujuan Qu

**Affiliations:** ^1^ Department of Medical Oncology, the First Hospital of China Medical University, Shenyang, China; ^2^ Key Laboratory of Anticancer Drugs and Biotherapy of Liaoning Province, the First Hospital of China Medical University, Shenyang, China; ^3^ Liaoning Province Clinical Research Center for Cancer, the First Hospital of China Medical University, Shenyang, China; ^4^ Clinical Cancer Treatment and Research Center of Shenyang, the First Hospital of China Medical University, Shenyang, China

**Keywords:** HAIC, immunotherapy, targeted therapy, subgroup analysis, sorafenib

## Abstract

**Background:**

The first-line treatment for advanced hepatocellular carcinoma has evolved significantly. This study aimed to identify the most beneficial regimen.

**Methods:**

A systematic search was conducted from July 2012 to August 2024 across the following four databases: PubMed, Embase, Cochrane Library, and ClinicalTrials.gov. This search focused on phase III prospective randomized controlled trials that compared first-line treatment for advanced hepatocellular carcinoma.

**Results:**

Seventeen studies involving 10322 patients were included in this network meta-analysis. Of the studies we included, twelve studies were global multicenter clinical studies, four were initiated in China, and one was initiated in Korea. The results of our statistical analysis suggest that Hepatic artery infusion chemotherapy with oxaliplatin plus fluorouracil (HAIC-FO) demonstrated significant overall survival (OS) benefits compared with most treatments, including various immune checkpoint inhibitors (ICIs) and anti-vascular endothelial growth factor tyrosine kinase inhibitors (VEGF-TKIs). In terms of OS, HAIC had shown similar efficacy with sorafenib plus FOLFOX (HR, 0.88; 95% CI: 0.37-2.09) and transcatheter arterial chemoembolization (TACE) combined with lenvatinib (HR, 0.69; 95% CI: 0.30-1.56). Notably, immune-related treatments, such as ICIs combined with anti-VEGF therapies, also showed improved OS compared with anti-VEGF-TKIs alone. In terms of progression-free survival (PFS), HAIC-FO outperformed anti-VEGF-TKI monotherapy, ICI monotherapy, and several ICI combinations. However, it was not superior to lenvatinib plus TACE or lenvatinib plus pembrolizumab. Based on the Surface Under the Cumulative Ranking Curve (SUCRA) values, HAIC-FO was ranked the most effective in terms of OS (SUCRA = 0.961) and objective response rate (ORR) (SUCRA = 0.971). The results of the subgroup analysis suggested that HAIC-FO achieved the best OS benefit in the macrovascular invasion (MVI) and extrahepatic spread (EHS) subgroup (SUCRA = 0.99) and that tremelimumab combined with durvalumab achieved the best OS benefit in the Asian subgroup (SUCRA = 0.88).

**Conclusion:**

This systematic review and network meta-analysis suggest that HAIC-based therapies may become a potential first-line treatment option for advanced HCC, especially for patients in Mainland China with MVI and EHS. Additionally, immune-related treatments may be more suitable for Asian populations.

## Introduction

Hepatocellular carcinoma (HCC) is a leading causes of cancer-related deaths worldwide (1), with its incidence varying significantly across regions due to differing prevalence of risk factors like HBV, HCV, and non-alcoholic fatty liver disease (NAFLD) (2). In China, HBV infection is the predominant cause, whereas in Western countries, chronic HCV infection and rising cases of alcohol-related and obesity-driven NAFLD are major contributors (3, 4). The diverse epidemiology and complex etiology of HCC, compounded by its genomic and molecular heterogeneity, present substantial treatment challenges. The tumor’s highly vascular nature, driven by angiogenesis, and the liver’s unique microenvironment further complicate treatment, with conventional chemotherapy often proving ineffective (5). Moreover, the immunosuppressive tumor microenvironment, characterized by regulatory T cells, myeloid-derived suppressor cells, and tumor-associated macrophages, inhibits anti-tumor responses (6). Consequently, there is an urgent need for novel therapies that can effectively target both the tumor and its microenvironment.

The first-line treatment paradigm for advanced HCC has undergone significant changes in recent years. Based on the phase III SHARP trial, sorafenib has been the standard first-line treatment for advanced HCC since 2008 (7). However, the efficacy of sorafenib is limited, as patients often develop resistance quickly, necessitating the exploration of new treatments. Considering that the tumor microenvironment (TME) of HCC patients exhibits recurring gene expression characteristics that are more consistent among patients, targeting the TME may be a more effective strategy than focusing on tumor cells alone (8). Therefore, numerous innovative studies have been conducted to integrate immunotherapy into the treament of HCC based on targeted therapy, yielding promising results (9). Pivotal results from IMbrave150 and ORIENT-32 trials have demonstrated that a combination of immune checkpoint inhibitors (ICIs) and anti-vascular endothelial growth factor (VEGF) inhibitors can significantly improve OS and are well-tolerated (10, 11). Moreover, combined therapy with ICIs has also shown improved OS compared to sorafenib in the HIMALAYA trial (12, 13).

In recent years, combined with systematic treatment, HAIC yields survival benefits over sorafenib, especially for patients with high intrahepatic disease burden and portal vein invasion (14, 15). A 2019 prospective phase III study involving 247 Chinese patients compared sorafenib plus HAIC with sorafenib plus HAIC with sorafenib alone. The median OS was 13.37 months in the combination arm versus 7.13 months in the sorafenib arm (HR, 0.35; 95% CI, 0.26-0.48; P < 0.001), underscoring the clinical value of HAIC-based combination as a first-line treatment for advanced HCC (10). Additionally, a 2021 prospective phase III study in found that HAIC alone significantly improved OS compared to sorafenib alone, particularly in high-risk subsets (10.8 versus 5.7 months; HR, 0.343; 95% CI: 0.219-0.538; *P* < 0.001) (11). Furthermore, combining HAIC with anti-VEGF TKIs, ICIs, and anti-VEGF antibodies has demonstrated high response rates and favorable survival outcomes with manageable adverse effects (16–20).

However, despite the availability of numerous treatment regimens, identifying the most effective and safe approach remains challenging. The relative efficacy and safety of various first-line treatments are still in question, and current guidelines lack tailored recommendations. There is a significant gap in studies addressing patients with HCC from different etiological backgrounds, such as those with NAFLD or HCV infection. To address this, our network meta-analysis incorporated data from prospective phase III clinical trials to identify optimal first-line treatments for advanced HCC. Specifically, we conducted key subgroup analyses, including etiological classification, population-based differences in treatment responses, the impact of microvascular invasion (MVI) and extrahepatic spread (EHS), and the predictive value of PD-L1 expression for immunotherapy outcomes. These comprehensive analyses aim to provide clearer insights that can guide personalized treatment strategies and improve clinical outcomes for diverse HCC patient populations.

## Methods

### Data sources and searches

Based on the Preferred Reporting Items for Systematic Reviews and Meta-Analyses (PRISMA) guidelines, PubMed, Embase, Cochrane Central Register of Controlled Trials, and ClinicalTrials.gov were searched to identify phase III randomized controlled trials (RCTs) regarding first-line treatment options for advanced HCC from 2012 to 2024. We also conducted a search for the latest data presented at the American Society of Clinical Oncology (ASCO) conference. To confirm the final selection, the reference lists of all the available reviewers were checked manually. Two reviewers (L.J. and L.D.) independently performed literature searches. Detailed search strategies are provided in **Supplementary Material** (**Supplementary Table 1**).

### Study selection and data extraction

To identify the studies for assessment, the titles, abstracts, and keywords of every retrieved record were independently reviewed by two reviewers. Full articles and congress reports were additionally evaluated if the information suggested that the study was a phase 3 RCT contrasting first-line regimens for the treatment of advanced HCC.

Two independent reviewers extracted and crosschecked the data separately. All disagreements were resolved after a discussion with a third-party assessor to reach a final consensus. The latest or most informative publications were selected if multiple publications were retrieved from the same study. Data extraction was performed at the trial level without individual patient data, because of the lack of approachability. General information was derived, including the journal name, document title, publication date, author, and country. The following information was obtained for each selected article: (1) study details (year of publication, name of authors); (2) baseline characteristics of participants (sex, age, the proportion of HBV infection, the proportion of HCV infection, the proportion of people with alpha-fetoprotein [AFP] > 400, Eastern Cooperative Oncology Group [ECOG] score, Barcelona Clinic Liver Cancer [BCLC] stage, the proportion of people with macrovascular invasion [MVI] and extrahepatic spread [EHS]) (3) interventions and drug dosages. Outcome measures included OS, progression-free survival (PFS), and objective response rate (ORR) (**Table 1**).

**Table 1:** Baseline characteristics.

Study	Study phase	Study design	Intervention arm	Control arm	No.	Male (%)	HBV HCV (%)	AFP> 400	Asia (%)	ECOG(%)		BCLC (%)	MVI (%) I/C	EHS (%) I/C	Primary endpoints	mFLP	mOS (m) I/C	mPFS (m) I/C	ORR (%) I/C
										0	1								
					I/C	I/C	I/C	I/C	I/C	I/C		I/C	I/C	I/C			I/C	I/C	I/C
**Chen 2021** (14)	Phase III	Prospective	HAIC-FO: Oxaliplatin: 130mg/m^2^ Leucivorin: 200mg/m^2^ Fliorouracil: 400mg/m^2^+ 2400mg/m^2^ Q3W	sorafenib: 400mgbid	130/ 132	92.3/ 84.6	92.3/ 84.6 NG	NG	100/100	11.5 /10.6	63.8/ 72 **2:** 24.6/14.7	NG	68.5/ 62.9	NG	OS	12.2	13.9/ 8.2	NG	31.5/ 1.5
**Minke 2019** (15)	Phase III	Prospective	SoraHAIC	sorafenib: 400mgbid	125/ 122	88.8/ 91.8	49/73 NG	NG	100/100	9.6/ 7.4	63.2/ 68 **2:** 27.2/ 24.6	NG	33/47	31/38	OS	10	13.3/ 7.1	7.03/2.6	40.8/ 2.5
**LAUNCH**	Phase III	Prospective	Lenvatinib: 12mg, BW>= 60 8mg, BW<60 TACE	lenvatinib	170/168	81.8/18.2	87.1/85.7 2.4/ 3.6	48.8/ 51.8	100/100	52.4/ 58.9	47.6/41.0	**NG**	71.8/69.6	55.3/ 56.5	OS	17.0	17.8/ 11.5	10.6/ 6.4	45.9/ 20.8
Study	Study phase	Study design	Intervention arm	Control arm	No.	Male (%)	HBV HCV (%)	AFP> 400	Asia (%)	ECOG(%)		BCLC (%)	MVI (%)	EHS (%)	Primary endpoints	mFLP	mOS (m)	mPFS (m)	ORR (%)
										1	1								
					I/C	I/C	I/C	I/C	I/C	I/C		I/C	I/C	I/C			I/C	I/C	I/C
**STAH**	Phase III	Prospective	Sorafenib: 200– 400 mg bid TACE	sorafenib	170/169	80/ 87	78.8/71	NG	100/100	80/82.8	19.4/ 16.6	**B**: 22.9/26 **C:** 75.3/74	NG	NG	OS	14/ 18.7	12.8/ 10.8	5.2/3.6	11.8/ 5.9
**Richard 2020** (10)	Phase III	Prospective	Atezol: 1200mg Beva: 15mg/kg Q3W	sorafenib: 400mgbid	336/ 165	82/ 83	49/46 21/22	38/37	40/ 41	62/ 62	38/38	**B:** 15/16 **C:** 82/81	38/43	63/65	PFS/ OS	8.6	19.2/ 13.4	6.8/ 4.3	30/ 11
**Ghassan 2022** (12)	Phase III	Prospective	T:300mg D:1500mg Q4W	sorafenib: 400mgbid	393/ 389	83.2/ 86.6	31/31 28/ 26.7	36.9/ 31.9	39.7/40.1	62/ 62	38/38	**B:** 19.6/ 17 **C:** 80.4/ 83	26.2/ 25.7	NG	OS	32.23	16.4/ 13.8	3.78/4.07	20.1/ 5.1
**Renz 2021** (11)	Phase III	Prospective	Sintilimab: 200mg Beva: 15mg/kg Q3W	sorafenib: 400mgbid	380/ 191	87.9/89.5	94.5/ 93.7 1.6/ 4.2	43.4/ 43.2	100/100	48/ 47.6	51.8/ 52.4	**B:** 14.7/ 14.1 **C:** 85.3/ 85.9	27.6/ 26.2	73.4/ 75.4	OS/ PFS	10	NG /10.4	4.6/ 2.8	20.5/ 4.1
**Cosmic** **312**	Phase III	Prospective	Cabozantinib: 40mgqd Atezol: 1200mg Q3W	sorafenib: 400mgbid	250/ 122	65/ 54	30/29 28/28	NG	27/ 30	65/ 61	35/ 39	**B:**33/34 **C:** 67/76	34/31	54/47	OS/PFS	15.8	6.8/4.2	15.4/15.5	13/5
Study	Study phase	Study design	Intervention arm	Control arm	No.	Male (%)	HBV HCV (%)	AFP> 400	Asia (%)	ECOG(%)		BCLC (%)	MVI (%)	EHS (%)	Primary endpoints	mFLP	mOS (m)	mPFS (m)	ORR (%)
										0	1								
					I/C	I/C	I/C	I/C	I/C	I/C		I/C	I/C	I/C			I/C	I/C	I/C
**CARES-310**	Phase III	Prospective	Cam: 200mg Q2W Apatinib: 250mg QD	sorafenib: 400mg bid	272/ 271	58/ 56	76.5/ 72.2 8.1/ 10.7	35.3/ 36.9	82.7/82.7	44.1/ 42.8	55.9/ 57.2	**B:** 14/ 14.8 **C：** 86/ 85.2	14.7/ 19.2	64.3/ 66.4	OS	NG	22.1/ 15.2	5.6/ 3.7	25.3/ 5.9
**Xu 2024 (****23****)**	Phase III	Prospective	**SCT-l10A:** **200mg** **Q3W** **SCT510:** **15mg/kg** **Q3Q**	**sorafenib:** **400mg** **bid**	**230/** **116**	**85.7/** **89.7**	**90.4/** **86.2**	**47.4/** **47.4**	**100/100**	**45.7/** **45.7**	**54.3/** **54.3**	**B:** **20/** **19.8** **C：** **80/** **80.2**	**37/44**	**61.7/** **59.5**	**OS/PFS**		**22.1/** **14.2**	**7.1/** **2.9**	**32.8/** **4.3**
Study	Study phase	Study design	Intervention arm	Control arm	No.	Male (%)	HBV HCV	AFP> 400	Asia (%)	ECOG(%)		BCLC (%)	MVI (%)	EHS (%)	Primary endpoints	mFLP	mOS (m)	mPFS (m)	ORR (%)
										0	1								
					I/C	I/C	I/C	I/C	I/C	I/C		I/C	I/C	I/C			I/C	I/C	I/C
**Kudo 2018** (26)	Phase III	Prospective	Lenvatinib: 12mg, BW>= 60 8mg, BW<60	sorafenib: 400mgbid	478/ 476	85/ 84	53/48 19/26	NG	70/ 68	64/ 63	38/36	**B:** 22/19 **C:** 78/81	23/19	61/62	OS	27.2	13.6/ 12/3	7.4/ 3.7	24.1/ 9.2
**Calin 2015** (27)	Phase III	Prospective	Linifanib: 17.5mg qd	sorafenib: 400mgbid	514/ 521	86.4/83.7	86.4/ 83.7 25.3/24.8	84.8/ 86.3	58.2/58.7	62.8/66.2	37.2/ 32.8	**B:** 15.8/ 19.6 **C:** 84.2/80.4	46.3/ 40.5	59.7/ 56.8	OS	NG	9.1/9.8	4.2/ 2.9	10.1/ 6.1
**Philip 2013** (28)	Phase III	Prospective	Brivanib: 800mg bid	sorafenib: 400mgbid	577/ 578	84/ 84	44/45 20/21	NG	64/ 60	61/ 64	39/ 36	**B:** 17/17 **C:** 78/77	27/27	NG	OS	NG	9.5/9.9	NG	12.0/ 9.0
**Cheng** **2013** (29)	Phase III	Prospective	Sunitinib: 37.5mg qd	sorafenib: 400mgbid	530/ 544	82.3/ 84.4	54.7/ 66.9 21.3/16.2	NG	77.5/100	52.5/51.5	46.8/ 48.3	**B:** 12.6/ 10 **C:** 87.2/ 90	78.9/ 83.3	NG	OS	7.8	7.9/ 10..2	3.6/ 3.0	6.6/ 6.0
Study	Study phase	Study design	Intervention arm	Control arm	No.	Male (%)	HBV HCV	AFP> 400	Asia (%)	ECOG(%)		BCLC (%)	MVI (%)	EHS (%)	Primary endpoints	mFLP	mOS (m)	mPFS (m)	ORR (%)
										0	1								
					I/C	I/C	I/C	I/C	I/C	I/C		I/C	I/C	I/C			I/C	I/C	I/C
**RATIONALE-301**	Phase III	Prospective	Tis: 200mg Q3W	sorafenib: 400mgbid	342/ 333	62/ 60	59.4/ 63 13.5/ 11.7	39.5/ 34.9	62.9/63.2	53.5/ 54.5	46.5/ 45.5	**B:** 20.5/ 24.1 **C:** 79.5/ 75.7	64/ 59.6	14.9/ 14.8	OS	NG	15.9/ 14.1	2.1/ 3.4	14.3/ 5.4
**Thomas 2021** (24)	Phase III	Prospective	Nivolumab: 240mg Q2W	sorafenib: 400mgbid	371/ 372	85/ 85	31/31 23/23	33/38	54/ 53	73/ 70	27/30	**B:** 14/17 **C:** 82/78	33/32	60/56	OS	15.2	16.4/14.7	3.7/ 3.8	7/15
**LEAP-002**	Phase III	Prospective	Lenvatinib 12mg, BW>= 60 8mg, BW<60 Pembrolizumab 200mg Q3W	Lenvatinib 12mg, BW>= 60 8mg, BW<60	395/399	80/ 82	49/49 24/22	30/ 33	31/ 31	68/68	32/32	**B:** 22/24 **C:** 78/76	18/16	63/61	OS	32.1	21.2/ 19	8.2/8.0	26.1/ 17.5

Baseline characteristics for patients included in the network meta-analysis.

atezol, atezolizumab; BCLC, Barcelona Clinic Liver Cancer; beva, bevacizumab; bid, twice a day; BM, body weight; Cabo, cabozantinib; Cam, camrelizumab; D, durvalumab; ECOG, Eastern Cooperative Oncology Group; EHS, Extrahepatic Spread; I/C, interventional arm/control arm: the left represents the proportion of the number of people in the intervention group, and the right represents the proportion of the number of people in the control group; mFLP, median follow up time; mOS, median overall survival; mPFS, median progression free survival; MVI, Macrovascular invasion; NG, not given; No., Number; ORR, objective response rate; Q2W, once every two weeks; Q3W, once every three weeks; qd, once a day; T, tremelimumab; TACE, transcatheter arterial chemoembolization; TIS, tislelizumab.

### Quality assessment

Bias was assessed using the Cochrane Collaboration’s Trial Bias Risk Assessment Tool, which includes the following: random sequence generation, concealment of allocation, blinding of participants and personnel, blinding of outcome assessments, incomplete outcome data, selective reporting, and other biases (**Supplementary Figure 1**). Two examiners evaluated the studies independently, and third-party authors resolved any disagreements.

### Statistical analysis

**Figure 1:**
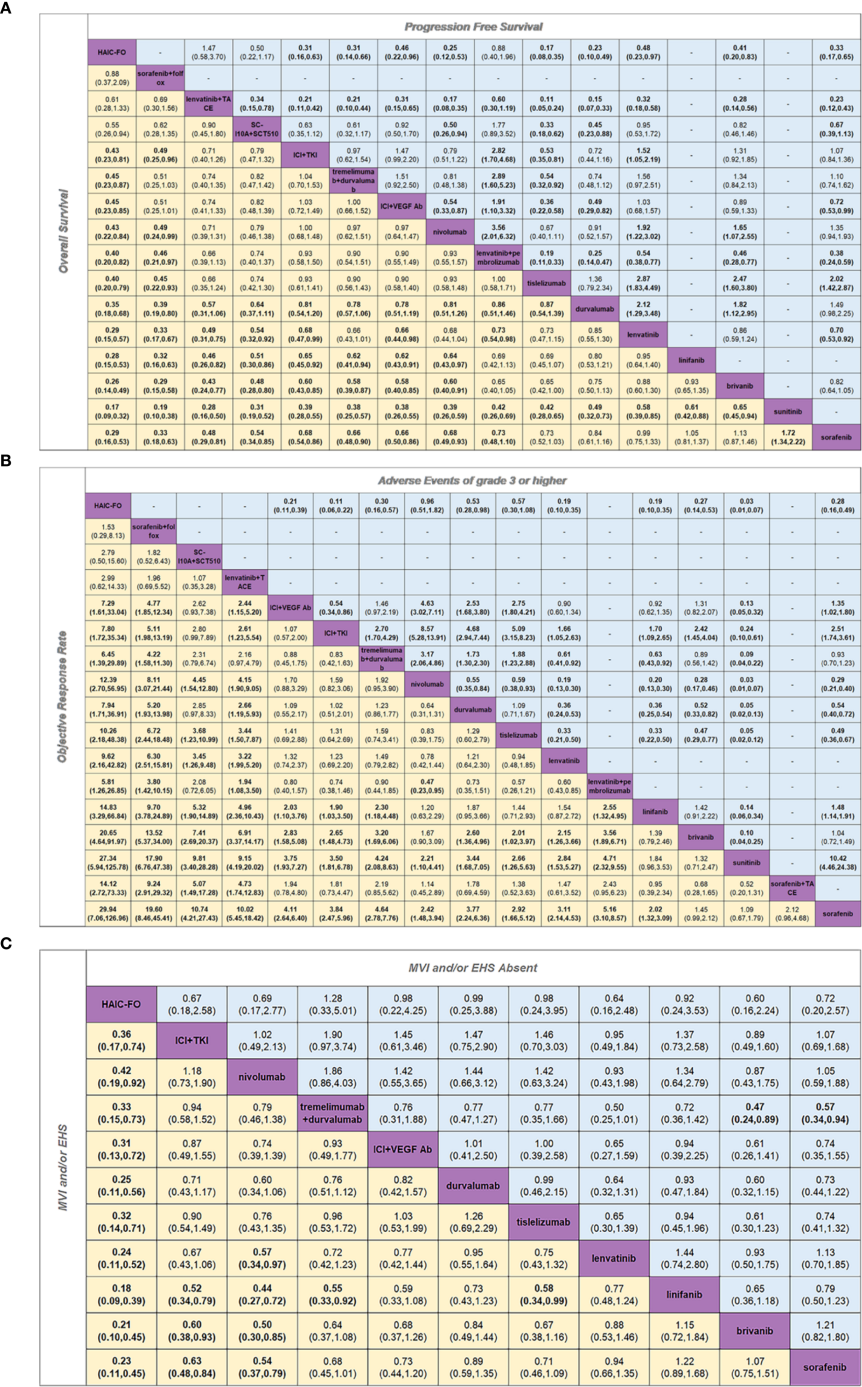
Pooled estimates of the network meta-analysis. **(A)** Pooled HR for PFS and OS. **(B)** Pooled OR for AEs of grade 3 or higher and ORR. **(C)** Pooled HR for MVI and/or EHS Absent subgroup and MVI and/or EHS subgroup. **(A)** Pooled HR (95% credible interval) for PFS (upper triangle) and OS (lower triangle). **(B)** Pooled OR (95% credible interval) for AEs of grade 3 or higher (upper triangle) and ORR (lower triangle). **(C)** Pooled HR (95% credible intervals) for the MVI and/or EHS Absent subgroup (upper triangle) and the MVI and/or EHS subgroup (lower triangle). Each cell contained an HR or OR (95% credible intervals) comparing the row-defining treatment to the column-defining treatment. Comparisons are shown from left to right. For OS, an HR of < 1 favors column-defining treatment. For PFS, an HR of less than one favors row-defining treatment. For ORR, an OR of > 1 favors column-defining treatment. For AEs of grade 3 or higher, an HR of < 1 favors row-defining treatment. For the MVI and/or EHS subgroups, an HR of < 1 favors row-defining treatment. For the MVI and/or EHS absent subgroup, an HR of less than one favors column-defining treatment. The significant results are shown in bold.

**Figure 2:**
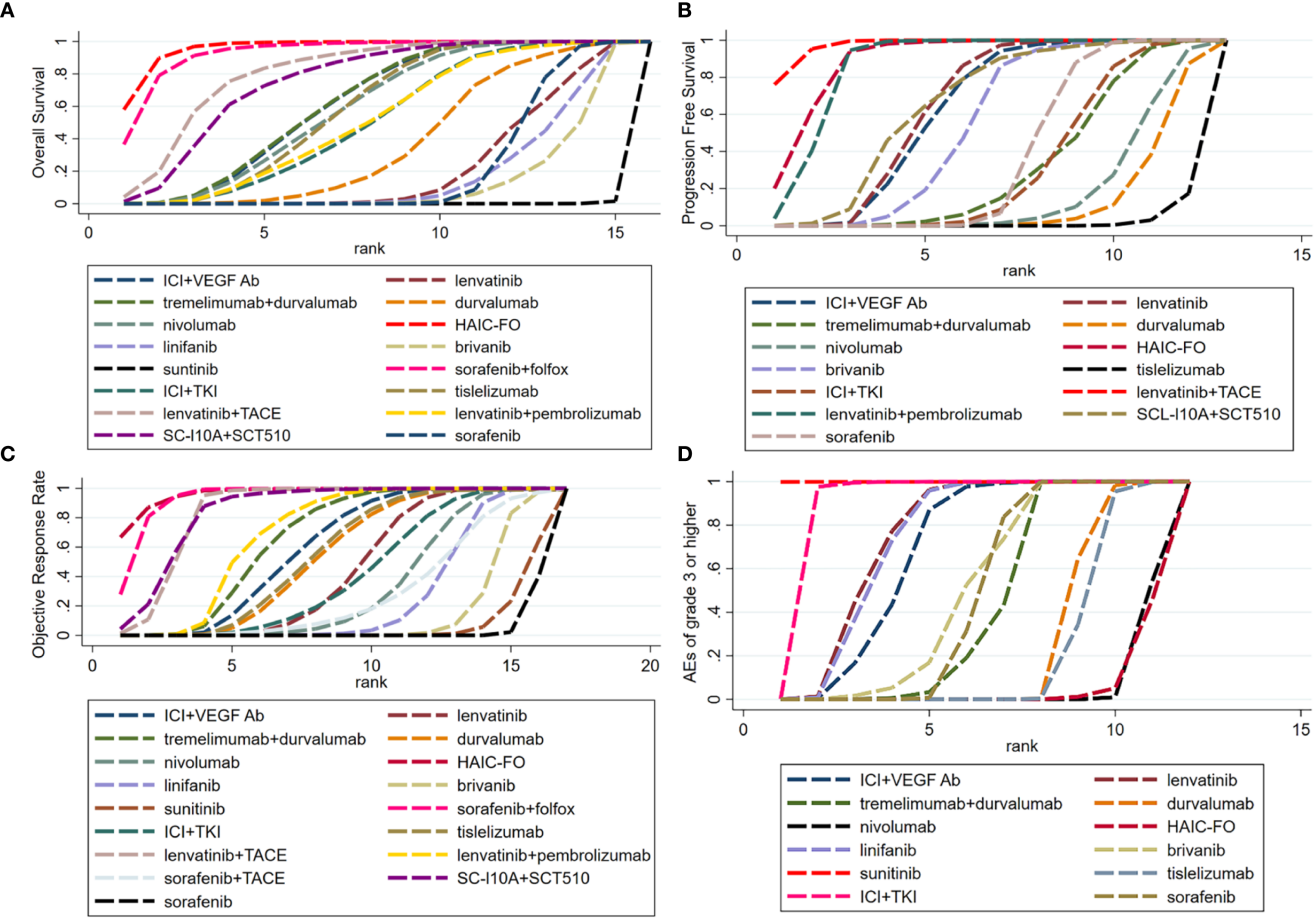
Ranking curves displaying the probabilities of **(A)** Overall Survival, **(B)** Progression Free Survival, **(C)** Objective Response Rate and **(D)** Adverse events of grade 3 or higher for each treatment. **(A)** Overall Survival. **(B)** Progression Free Survival. **(C)** Objective Response Rate. **(D)** Adverse events of grade 3 or higher. 3**(A)** Ranking curves of OS **(B)** Ranking curves of PSF **(C)** Ranking curves of ORR **(D)** Ranking curves of AEs ≥ grade 3. Bayesian ranking profiles of comparable treatments for efficacy in patients with advanced HCC. Profiles indicate the probability of each comparable treatment being ranked first to last on OS and PSF.

To assess outcomes and safety, hazard ratios (HR) for survival outcomes (OS and PFS), OR, or binary outcomes (ORR and TRAEs grade 3 or higher), and their 95% CIs were calculated. First, network diagrams were plotted to visualize all therapies, with the size of the dots representing the sample size and the breadth of the line representing the quantity of similar included studies (**Supplementary Figures 2, 3**). Second, ladder diagrams were also created, which could intuitively reflect the HR or OR value between any two interventions (**Figures 1**; **Supplementary Figures 4, 5**). Third, the surface under the cumulative ranking curve (SUCRA) values for all regimens were calculated, representing the probability that a regimen was ranked best, used to evaluate the effectiveness of each intervention (**Figure 2**; **Supplementary Figure 8**). Heterogeneity between studies was assessed using a Q test and *I* ^2^ statistic (**Supplementary Figure 6**), with an estimated *I* ^2^ of less than 25% considered low, between 25% and 50% considered moderate, and more than 50% considered high. Sensitivity analysis was conducted to test the stability of the model (**Supplementary Figure 7**). Finally, given that the included studies span over a decade, we performed a stratified analysis that specifically accounts for the time factor, focusing on the primary endpoint of overall survival (OS) across all studies (**Supplementary Figure 9**). All statistical analyses were performed using Stata software (version 15.1) using a random effects model. The included studies did not form a ring structure; therefore, we did not conduct an inconsistency test.

## Results

### Study selection and characteristics

**Figure 3:**
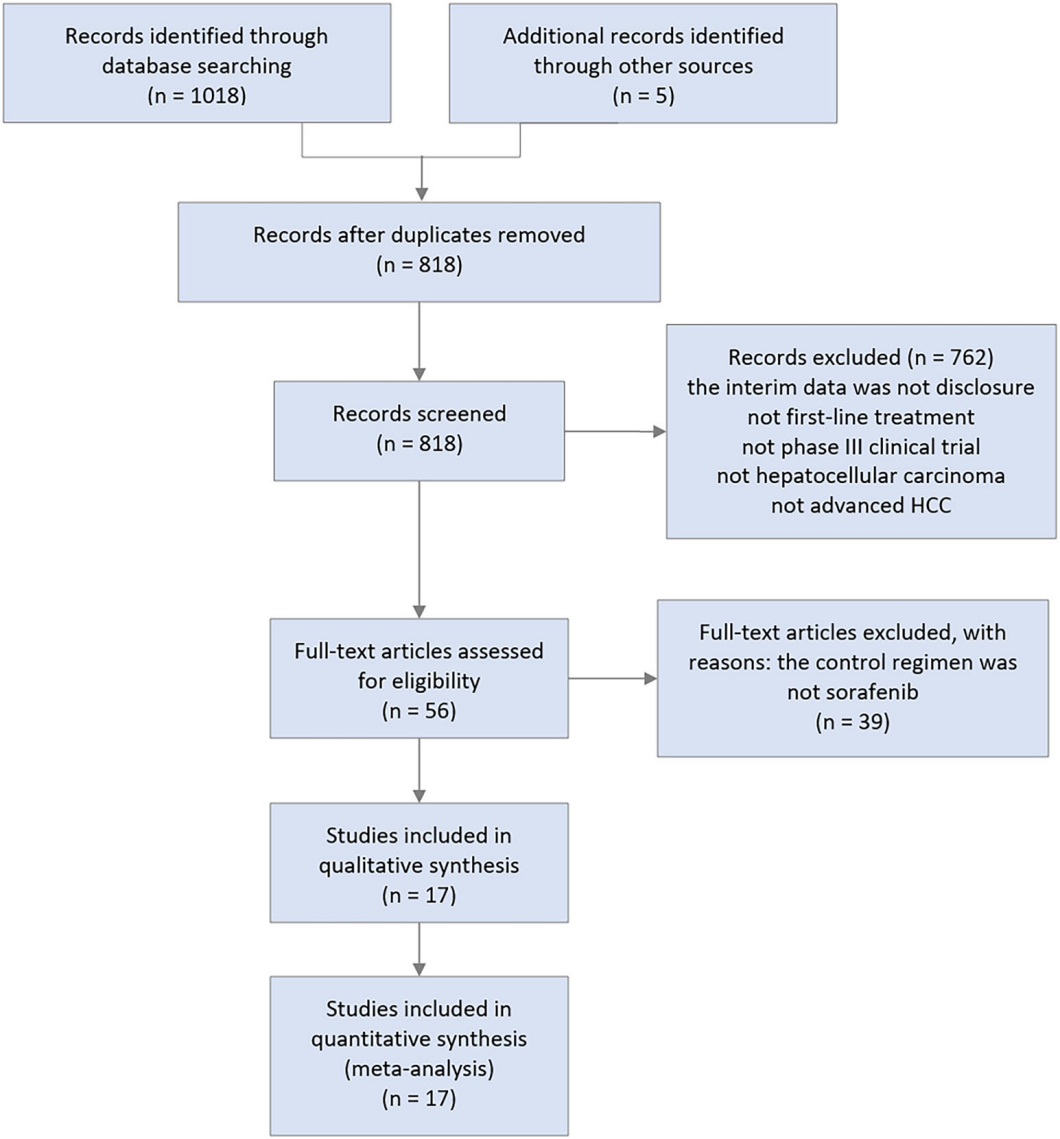
Flowchart of study selection.

The Preferred Reporting Items for Systematic Reviews and Meta-Analyses checklist for our meta-analysis is provided in **Supplementary Data Sheet 2**. A total of 1,018 records were identified from the initial screening of titles and abstracts. Finally, 17 RCTs were eligible for inclusion, with 10322 enrolled patients receiving 17 different treatments (**Figure 3**), including targeted therapy combined with immunotherapy (10, 11, 21–23), immunotherapy combined with immunotherapy (9), immunotherapy monotherapy (24, 25), tyrosine kinase inhibitors (26–29), TACE-based treatments (30, 31), and HAIC-based treatments (10, 11). A summary of the main characteristics and results of the included studies is shown in **Table 1**.

### Quality assessment and risk of bias

Each study was qualitatively evaluated by estimating different indicators using the Cochrane bias tool. Overall, the trials were considered to have a low risk of bias, except for detection and performance bias, in which only one study blinded the results, whereas the remaining studies did not. (**Supplementary Figure 1**).

### Overall survival

Based on the results of this network meta-analysis in terms of OS (**Figure 1A**), Hepatic artery infusion chemotherapy with oxaliplatin plus fluorouracil (HAIC-FO) treatment showed significant benefits compared to almost all other treatments including ICIs plus anti-VEGF, TKIs (HR, 0.45; 95% CI 0.23-0.86), ICIs plus ICIs (HR, 0.45; 95% CI, 0.23-0.87), ICIs plus anti-VEGF antibody (HR, 0.45; 95% CI 0.23-0.86), SC-L10A plus SCT510 (HR, 0.55; 95% CI,0.26-0.94), nivolumab (HR, 0.43; 95% CI, 0.22-0.84), lenvatinib plus pembrolizumab (HR, 0.40; 95% CI, 0.20-0.83), tislelizumab (HR, 0.40; 95% CI, 0.20-0.79), durvalumab (HR, 0.35; 95% CI, 0.18-0.68), lenvatinib (HR, 0.29; 95% CI, 0.15-0.57), linifanib (HR, 0.28; 95% CI, 0.15-0.53), brivanib (HR, 0.26; 95% CI, 0.14-0.49), sunitinib (HR, 0.17; 95% CI, 0.09-0.32) and sorafenib (HR, 0.29; 95% CI, 0.16-0.53), except for sorafenib plus FOLFOX (HR, 0.88; 95% CI, 0.37-2.09) and transcatheter arterial chemoembolization (TACE) combined with lenvatinib (HR, 0.61; 95% CI, 0.28-1.33). It is worth noting that immune-related treatments, including ICI plus anti-VEGF antibody, ICI plus anti-VEGF TKIs, tremelimumab plus durvalumab and nivolumab, also showed meaningful OS improvement compared with anti-VEGF TKIs. However, significant heterogeneity was observed in the heterogeneity test (*I* ^2^ = 83.9%, *P* = 0.000).

### Progression-free survival

Regarding PFS (**Figure 1A**), HAIC-FO demonstrated superior efficacy compared to all anti-VEGF TKI monotherapies, including lenvatinib (HR, 0.48; 95% CI, 0.23-0.97), brivanib (HR, 0.41; 95% CI, 0.20-0.83), and sorafenib (HR, 0.33; 95% CI, 0.17-0.65), and showed significant benefits compared to ICI monotherapy: durvalumab (HR, 0.23; 95% CI, 0.10-0.49), nivolumab (HR, 0.25; 95% CI, 0.12-0.53), and tislelizumab (HR, 0.17; 95% CI, 0.08-0.35). Furthermore, HAIC-FO outperformed various ICI combinations, including ICI plus VEGF Ab (HR, 0.46; 95% CI, 0.22-0.96), ICI plus anti-VEGF TKIs (HR, 0.31; 95% CI, 0.16-0.63), and tremelimumab plus durvalumab (HR, 0.31; 95% CI, 0.14-0.66). However, HAIC-FO was not superior to lenvatinib plus TACE (HR, 1.47; 95% CI, 0.58-3.70), SC-L10A plus SCT510 (HR, 0.63; 95% CI, 0.28-1.40) and lenvatinib plus pembrolizumab (HR, 0.88; 95% CI, 0.40-1.96). The studies showed significant heterogeneity (*I^2^* = 84.5%, *P* = 0.000).

### Objective response rate

Regarding ORR (**Figure 1B**), we found that in addition to brivanib (HR, 1.45; 95% CI, 0.99-2.12), sunitinib (HR, 1.09; 95% CI, 0.67-1.79) and sorafenib combined with TACE (HR, 2.12; 95% CI, 0.96-4.68) all other regimens achieved significant ORR benefits compared with sorafenib. HAIC-based treatment achieved encouraging ORR benefits over nearly all the other regimens. Similarly, there was no significant difference between HAIC-FO, sorafenib combined with FOLFOX (HR, 1.53; 95% CI, 0.29-8.13), lenvatinib combined with TACE (HR, 2.99; 95% CI, 0.62-14.33) and SC-L10A plus SCT510 (HR, 2.79; 95% CI, 0.50-15.6). In addition to HAIC-based treatment, combination therapies have achieved significant ORR benefits, including ICls combined with ICls (SUCRA = 0.687), a combination of ICls and anti-VEGF antibody (SUCRA = 0.622), and ICIs combined with anti-VEGF TKIs (SUCRA = 0.584). The studies showed significant heterogeneity (*I* ^2^ = 80.8%, *P* = 0.000).

### Adverse events of grade 3 or higher and treatment-related serious adverse events

We assessed the safety of different treatment options and found that HAIC-FO had the lowest probability of causing adverse events (AEs) of grade 3 or higher, and was least likely to be ranked first (SUCRA = 0.047). Tislelizumab and nivolumab had consistent safety profiles compared to HAIC-FO (tislelizumab: HR, 0.57; 95% CI, 0.30-1.08; nivolumab: HR, 0.96; 95% CI, 0.51-1.82). Among all regimens, sunitinib had the highest risk of causing grade ≥ 3 AEs (SUCRA = 1) (**Supplementary Table 2**). However, we observed significant heterogeneity across studies (*I* ^2^ = 95.1%, *P* = 0.000). When considering treatment-related serious adverse events (TRSAEs) (**Supplementary Figure 4**), the combination of ICI plus anti-VEGF TKIs had the highest probability of causing the most TRSAEs, with significant heterogeneity detected among the studies (*I* ^2^ = 80.4%, *P* = 0.000).

### HBV, HCV, and nonviral

In the population infected with HBV (shown in **Supplementary Figure 5A**), sorafenib and HAIC of FOLFOX demonstrated OS benefits over almost all regimens, except for HAIC-FO (HR, 0.57; 95% CI, 0.21-1.52), tremelimumab plus durvalumab (HR, 0.42; 95% CI, 0.16-1.11) and lenvatinib plus TACE (HR, 0.37; 95% CI, 0.14-0.99). Additionally, we found that ICIs combinations, except for ICI plus VEGF Abs, showed superior efficacy compared to anti-VEGF TKIs monotherapies. This subgroup showed significant heterogeneity (*I* ^2^ = 80%, *P* = 0.000).

In the HCV-infected population, we found that only ICI plus anti-VEGF TKIs was more proficient with sorafenib (HR, 0.38; 95% CI, 0.16-0.88) regarding OS, reflected by ICI plus anti-VEGF TKIs ranking the highest in terms of OS in the HBV subgroup. No significant differences were observed between regimens. No significant heterogeneity was found in this subgroup (*I* ^2^ = 53.2%, *P* = 0.036).

In the non-viral infected population (**Supplementary Figure 5C**), with significant heterogeneity of HAIC resulting from the limited sample size (**Supplementary Figure 6F**), tremelimumab plus durvalumab was associated with better OS. No significant heterogeneity was observed across studies (*I^2^* = 17.1%, *P* = 0.289).

### Asia and non-Asian

In the Asian subgroup (**Supplementary Figure 5D**), tremelimumab plus durvalumab (HR, 0.52; 95% CI, 0.37-0.81), nivolumab (HR, 0.57; 95% CI, 0.35-0.93), and ICI plus anti-VEGF TKIs were associated with better OS benefits than other therapies. No significant difference in OS benefit was found among the treatments in the non-Asian group (**Supplementary Figure 5E**). We found substantial heterogeneity among the studies in the analysis of the Asian group (*I* ^2^ = 82.1%, *P* = 0.000), whereas no significant heterogeneity was found in non-Asian populations (*I* ^2^ = 6.5%, *P* = 0.381).

### PD-L1 positive and negative

We did not find any difference between the various treatments in either PD-L1 expression-positive (**Supplementary Figure 5F**) or-negative (**Supplementary Figure 5G**) subgroups. Moreover, no heterogeneity was observed in PD-L1 positive subgroup (*I* ^2^ = 0.0%, *P* = 0.706) or PD-L1 negative subgroup (*I* ^2^ = 0, *P* = 0.556).

### MVI and/or EHS, MVI and/or EHS absent

For the MVI and/or EHS subgroups (**Figure 1C**), we observed significant OS benefits in HAIC-FO versus all the remaining treatments, including ICI plus anti-vascular TKI (HR, 0.36; 95% CI, 0.17-0.74), ICIs combined with ICIs (HR, 0.33; 95% CI, 0.15-0.73), ICI plus VEGF Ab (HR, 0.31; 95% CI, 0.13-0.72), nivolumab (HR, 0.42; 95% CI, 0.19-0.92), durvalumab (HR, 0.25; 95% CI, 0.11-0.56), tislelizumab (HR, 0.32; 95% CI, 0.14-0.71), lenvatinib (HR, 0.24; 95% CI, 0.11-0.52), linifanib (HR, 0.18; 95% CI, 0.09-0.39), brivanib (HR, 0.21; 95% CI, 0.10-0.45), and sorafenib (HR, 0.23; 95% CI, 0.11-0.45). Among these, HAIC-FO is the optimal treatment option. Moreover, nivolumab and ICI plus anti-vascular TKI have shown increased efficacy. We found no significant heterogeneity in this subgroup (*I* ^2^ = 75%, *P* = 0.000).

In the MVI and/or EHS absent subgroup (**Figure 1C**), we found no significant differences in OS benefit among the various treatments, except for tremelimumab plus durvalumab, which showed a significant advantage compared with brivanib (HR, 0.47; 95% CI, 0.24-0.89) and sorafenib (HR, 0.57; 95% CI, 0.34-0.94). Comparison between the MVI and/or EHS and MVI and/or EHS absent subgroups showed more pronounced differences in OS benefits with HAIC-FO in the MVI/EHS subgroup. No heterogeneity was observed in this subgroup (*I* ^2^ = 0, *P* = 0.467).

### Rank probabilities

Using the Bayesian ranking algorithm, we used the SUCRA value to rank the various treatment options for different populations, as shown in **Supplementary Table 2**. The surface under the cumulative ranking curve SUCRA metric was used to rank the effectiveness or safety of each treatment and identify the best treatment (**Figure 2**). A higher SUCRA value indicated a higher probability of the treatment regimen being ranked first. Our findings suggest that HAIC-FO was most likely to be ranked first in terms of OS (**Figure 2A**, SUCRA = 0.962), ORR (**Figure 2C**, SUCRA = 0.965), and MVI and/or EHS subgroups (**Supplementary Figure 8F**, SUCRA = 0.99). Sorafenib plus HAIC with FOLFOX was most likely ranked first in the HBV infection subgroup (**Supplementary Figure 8C**, SUCRA = 0.982). In the HCV Infection subgroup (**Supplementary Figure 8D**), ICI plus VEGF Ab was most likely to be ranked first (SUCRA = 0.875). In the non-viral infection subgroup, HAIC-FO was most likely to be ranked first (**Supplementary Figure 8E**, SUCRA = 0.964).

Tremelimumab plus durvalumab had the highest probability of being ranked first in the Asian subgroup (**Supplementary Figure 8A**, SUCRA = 0.88) and MVI and/or EHS Absent subgroup (**Supplementary Figure 8G**, SUCRA = 0.864). In the non-Asian subgroup, tislelizumab had the highest probability of ranking first (**Supplementary Figure 8B**, SUCRA = 0.83). In the PD-L1 positive subgroup, ICI plus anti-VEGF Ab had the highest probability of ranking first (**Supplementary Figure 8H**, SUCRA = 0.812); in the PD-L1 negative subgroup, nivolumab was most likely to be ranked first (**Supplementary Figure 8I**, SUCRA = 0.768). Furthermore, HAIC-FO had the lowest probability of causing AEs of grade 3 or higher (**Figure 2D**, SUCRA = 0.047). Sorafenib plus Folfox had the lowest likelihood of causing TRSAEs (**Supplementary Figure 8J**, SUCRA = 0.163).

### Sensitivity analysis

In the overall population, in terms of efficacy and safety, we found that the 95% CI of the remaining studies was one after removing most of the studies, which verified the stability of the model (**Supplementary Figure 7**). However, in some subgroups, such as HCV infection, non-viral infection, MVI, and/or EHS absence, the models were less stable, possibly due to the uneven sample sizes included in these subgroups.

### Stratified analysis

The stratified analysis revealed that studies conducted before 2018 primarily reported negative outcomes. However, from 2018 onward, the introduction of immunotherapy and transarterial infusion therapies resulted in positive outcomes in phase III clinical trials for first-line treatment of advanced HCC compared to control groups (**Supplementary Figure 9**).

## Discussion

This study provides the most detailed and up-to-date comparison of first-line treatments for advanced HCC using network meta-analysis (NMA). The aim was to evaluate the efficacy and safety of various treatment options for for patients with advanced HCC, especially for those with microvascular invasion (MVI) and/or extrahepatic spread (EHS). Additionally, patients treated with HAIC-based regimens reported fewer severe AEs (grade ≥ 3) compared to those receiving other treatments. Currently, the number of prospective clinical studies on HAIC-based treatments is limited globally, with most studies being retrospective analyses or small-sample single-arm studies (20, 32, 33). Our research is based on prospective phase III study results to ensure better scientific rigor. The Chinese Society of Clinical Oncology recently endorsed HAIC, specifically with fluorouracil, oxaliplatin, and leucovorin, as a viable alternative for advanced HCC (34). Moreover, the Japanese HCC clinical practice guidelines recommend HAIC for patients with advanced HCC to improve prognosis (35). These findings support the potential of HAIC-based therapies as superior approaches for treating patients with advanced HCC. We conducted a network meta-analysis to validate this potential and identify the most effective regimen for various patient groups.

The results showed that HAIC-FO was associated with remarkably upgraded OS, PFS, and ORR benefits compared to other first-line agents, except for the regimen of sorafenib plus HAIC of FOLFOX. Furthermore, HAIC-FO had the lowest probability of causing AEs of grade 3 or higher. HAIC is believed to cause a higher intertumoral concentration of chemotherapy agents while avoiding the first-pass effect, thereby improving efficacy and reducing liver toxicity (36). These mechanisms may partly explain why patients with HCC and MVI/EHS could benefit most from HAIC. Additionally, chemotherapeutic agents pass through the body’s circulation and offer a systematic antitumor effect at lower concentrations, thus causing fewer side effects.

Previous studies have consistently shown that HAIC-based treatments significantly improve the ORR in patients, implying that HAIC-based therapy may offer the possibility of conversion therapy for advanced HCC. One phase II retrospective trial found that a combination of HAIC, oral antiangiogenic drugs, and programmed death-1 inhibitors achieved a 96% ORR and 60% surgical conversion rate (37). In addition, a current retrospective phase II trial evaluated the effectiveness of a triple combination of lenvatinib, anti-PD-1 antibodies, and transarterial therapy for unresectable HCC, which resulted in an ORR of 67.7% and conversion rate of 40.5% (14). Furthermore, the combination of HAIC with sintilimab and bevacizumab biosimilar (IBI-305) as the first-line treatment showed a confirmed ORR of 66.7%, with a conversion rate of 66.7% for patients with advanced HCC (17). A phase II clinical study showed HAIC-FO plus camrelizumab plus apatinib showed a confirmed ORR of 77.1% (20). The promising results regarding ORR, conversion rate, and survival benefits of HAIC-based treatment, along with its manageable safety profile, indicated that HAIC-based combination therapy would emerge as the prevalent tendency in clinical practice and clinical trials. Besides HAIC, TACE, as a local treatment method, also plays a crucial role in the tumor transformation benefits for advanced HCC. A retrospective analysis revealed that combining TACE, PD-(L1) inhibitors, and molecularly targeted treatments (MTT) in HCC resulted in a confirmed ORR of 60.1%, notably superior to TACE monotherapy (ORR: 32%) (38). Another retrospective analysis showed that TACE plus camrelizumab and apatinib yielded an ORR of 59.5 (39).

In addition to HAIC-based therapy, immunotherapy plays a crucial role in different subgroups. Patients with HBV infection benefited more from HAIC-based treatment in terms of OS. Additionally, ICI combinations—except those paired with VEGF antibodies—demonstrated superior efficacy compared to anti-VEGF-TKI monotherapies. Previous studies demonstrated that HBV-infected HCC patients are more responsive to ICIs due to ongoing inflammation, which drives immune checkpoint expression and T-cell exhaustion (40). Our study found that HAIC-based therapy resulted in a superior OS benefit in patients with HBV infection. However, Zhao et al. reported that HBV reactivation occurred more frequently in HBsAg-positive patients receiving HAIC without antiviral therapy (41), highlighting the need for vigilant HBV DNA monitoring and antiviral prophylaxis during immunotherapy. Given the comparable efficacy of ICI combinations to HAIC, they may be recommended for HBV-infected patients prioritizing safety. Additionally, in the Asian and MVI/EHS subgroups, therapies such as tremelimumab plus durvalumab, nivolumab, and ICI plus anti-VEGF TKIs demonstrated superior OS benefits over sorafenib. However, the predictive effect of PD-L1 expression on immunotherapy in HCC remains unclear, as our study confirmed. Similarly, HCV infection is a major cause of HCC, requiring tailored therapeutic approaches. Our subgroup analysis suggests that ICI combined with anti-VEGF antibody may be more suitable for this group of patients. However, our analysis is based solely on the currently available subgroup data. In fact, among the 17 studies included, only 8 provided data on patients with HCV infection. Therefore, the choice of treatment regimens for this patient population still requires more prospective clinical research to verify. Unlike HBV infection, the progression from HCV infection to HCC involves a long-term chronic inflammatory process. HCV patients are 10 to 20 times more likely to develop liver cirrhosis than those with HBV, making it essential to assess their liver function when selecting anticancer drugs (42). Timely treatment and intervention are crucial for these patients. While direct-acting antiviral (DAA) drugs have greatly improved HCV treatment, some HCV subtypes exhibit genetic polymorphisms that lead to high resistance to DAAs, presenting substantial challenges for managing this patient group (3).

In fact, the heterogeneity of liver cancer caused by different etiologies, such as HBV and HCV infections, is also the primary reason for therapeutic resistance, posing a critical clinical challenge. Tumor heterogeneity in advanced HCC patients is often the HCC demonstrates profound heterogeneity at the genomic, epigenetic, transcriptional, and protein levels, contributing to chemotherapy ineffectiveness (43–46). Resistance to targeted therapy in HCC may result from alterations in cell signaling pathways, dysregulation of apoptosis or survival pathways, and changes in drug metabolism pathways (5). Additionally, factors such as microbiome diversity, metabolism alterations, tumor immune environment and immune evasion are linked to resistance to immunotherapy (9, 47). To bridge basic and clinical research, we need to delve deeper into the molecular mechanisms underlying drug resistance and immune evasion to identify novel therapeutic targets. Moreover, there should be a heightened focus on preclinical biomarker discovery. For instance, liquid biopsy is emerging as a promising method for identifying immunotherapy-related markers to predict treatment response (48). This technique can assess the efficacy of immunotherapy by measuring changes in circulating tumor DNA, circulating tumor cells, lymphocyte subsets, exosomes, and metabolites (49). Furthermore, to enhance the predictive validity of preclinical findings and validate therapeutic approaches, it is essential to utilize advanced models such as patient-derived xenograftes and organs (50, 51).

This study has several limitations. First, the NMA approach has inherent limitations. Most evidence relies on indirect comparisons and assumptions of transitivity and consistency, despite including only randomized controlled trials and investigating these assumptions. Second, this study suffers from possible publication and selection bias. Furthermore, the use of research-grade data for analysis may limit its statistical power compared with individual patient data. Moreover, the results of the heterogeneity analysis indicated that inequalities in the study sample sizes may lead to increased heterogeneity. In addition to methodological limitations, the study of HAIC also has several limitations. Firstly, most studies compare HAIC only to sorafenib, limiting its evaluation against other standard first-line therapy treatments like atezolizumab plus bevacizumab. Additionally, the majority of participants are from Mainland China and primarily have HBV infection, raising concerns about the generalizability of the results to patients with other causes of HCC, such as HCV or alcohol-related liver disease. Finally, HAIC is also invasive and carries risks of complications, including catheter displacement and thrombosis. In the future, more global multicenter randomized controlled trials with different etiological backgrounds are needed to confirm the efficacy of HAIC in HCC patients. These studies will help improve our understanding of the applicability of HAIC across different patient populations. Therefore, it is essential to consider these limitations when interpreting the results. Nonetheless, this study provides valuable insight into the evolution of first-line therapies for advanced HCC.

## Conclusions

Our network meta-analysis suggests that HAIC-based regimens may prolong the survival of patients with advanced HCC, with a manageable safety profile.Immune-related therapies also show promise, particularly within the Asian subgroup. Future research should focus on larger, global multicentre randomized controlled trials to provide more robust evidence and confirm the global applicability of HAIC-based strategies.

## Data Availability

The original contributions presented in the study are included in the article/**Supplementary Material**. Further inquiries can be directed to the corresponding authors.
