# Supplementary material for: Efficacy and safety of first-line treatments for advanced hepatocellular carcinoma patients: a systematic review and network meta-analysis
Source: Front Immunol. 2024 Sep 17;15:1430196. doi: 10.3389/fimmu.2024.1430196 (PMC11442238; doi:10.3389/fimmu.2024.1430196)
Supplement: Supplementary file 1 [file DataSheet1.pdf]

Supplementary Online Content

eTable 1. Search Strategies
eTable 2. SUCRA Value for different regimens
eFigure 1. Risk of bias graph for advanced hepatocellular first-line treatments: review authors' judgements about each risk of bias item presented as percentages across all included studies
eFigure 2. Network diagrams of comparisons on different outcomes of treatments in patients with Advanced Hepatocellular Carcinoma
eFigure 3. Network diagrams of comparisons on OS of treatments in different subgroups of patients with Advanced Hepatocellular Carcinoma
eFigure 4. League table showing treatment-related serious adverse events
eFigure 5. League table showing subgroup analysis
eFigure 6. Forest plot of Frequentist network meta-analysis using random-effects model
eFigure 7. Analysis of sensitivity
eFigure 8. Ranking curves displaying the probabilities of different regimens
eFigure 9. Stratified analysis focusing specifically on the time factor

eTable 1. Search Strategies

Sources	Search strategies
Pubmed: 367 Results	(((("Carcinoma, Hepatocellular"[Mesh]) AND "Liver Neoplasms"[Mesh] OR Carcinomas, Hepatocellular OR Hepatocellular Carcinomas) AND (((("Drug Therapy"[Mesh]) OR "drug therapy" [Subheading]) OR "Immunotherapy"[Mesh]) OR "Protein Kinase Inhibitors" [Pharmacological Action])) AND (advanced[tiab] OR unresctable[tiab] OR metatas*[tiab] OR metastat*[tiab])) AND (Clinical Trial, [ptyp])
Embase: 415Results	(“Liver cancer”: ab,ti OR” liver cancer”:exp OR “hepatocellular carcinoma”:ab,ti) AND (“sorafenib”:ab,ti) AND(“randomized controlled trila”/exp) AND (“advanced”:ab,ti OR “unresctable”:ab,ti OR “metastatic”:ab,ti)
Corchrane: 215Results:	#1 Mesh descriptor: [Liver Neoplasms] explode all trees<br>#2 drug therapy<br>#3 Mesh descriptor: [Drug Therapy explode all trees]<br>#4 (drug therapy): ti,ab,kw<br>#5Mesh descriptor: [immunotherapy] explode all trees<br>#6(protein kinase inhibitors) ti,ab,kw<br>#7 #3 OR #4 OR #5 #6<br>#8(advanced): ti,ab,kw<br>#9(“unresctable”): ti,ab,kw<br>#10metatas*<br>#11 #8 OR #9 OR #10<br>#12 (Clinical Trial): ti,ab,kw<br>#13#1 AND #7 AND #11 AND #1

eTable 2. SUCRA Value for different regimens

A. SUCRA value of the whole population

**Treatment**	**SUCRA**				
	OS	PFS	ORR	AEs	TRSAEs
ICI+anti-VEGF Ab	0.615	0.623	0.584	0.677	0.583
Lenvatinib	0.22	0.645	0.433	0.745	0.712
Tremelimumab+durvalumab	0.62	0.313	0.65	0.423	0.783
Durvalumab	0.373	0.119	0.529	0.239	0.168
Nivolumab	0.586	0.17	0.317	0.051	-
HAIC-FO	**0.962**	**0.894**	**0.965**	**0.047**	-
Linifanib	0.177	-	0.244	0.733	-
Brivanib	0.133	0.542	0.137	0.50	-
Sunitinib	0.001	-	0.059	1	-
Sorafenib+folfox	0.93	-	0.939	-	**0.163**
Tislelizumab	0.51	0.017	0.407	0.209	0.373
ICI+anti-VEGF TKIs	0.582	0.314	0.549	0.907	0.991
Lenvatinib+TACE	0.807	0.976	0.848	-	-
Lenvatinib+pembrolizumab	0.516	0.865	0.685	-	-
Sorafenib+TACE	-	-	0.279	-	-
SC-l10A+SCT510	0.754	0.651	0.848		
Sorafenib	0.215	0.371	0.027	0.469	0.227

B. SUCRA value of different subgroup people

**Treatment**	**SUCRA of OS**				
	HBV	HCV	Nonviral	Asia	Nonasia
ICI+anti-VEGF Ab	0.447	**0.875**	0.125	0.58	0.66
Lenvatinib	0.202	0.293	-	0.43	0.16
Tremelimumab+durvalumab	0.765	0.186	0.783	**0.88**	0.66
Durvalumab	0.461	0.125	0.614	0.4	0.6
Nivolumab	0.508	0.713	0.537	0.82	0.64
HAIC-FO	**0.878**	-	**0.964**	-	-
Linifanib	0.178	-	-	0.13	0.69
Brivanib	0.253	0.698	-	0.18	0.12
Sorafenib+folfox	**0.982**	-	-	-	-
Tislelizumab	0.32	**0.851**	0.517	0.5	**0.83**
ICI+anti-VEGF TKIs	0.658	0.438	0.193	0.76	0.3
Lenvatinib+TACE	0.695	-	-	-	-
Sorafenib	0.155	0.321	0.267	0.29	0.29

eTable 2 (Continued)

B. SUCRA value of different subgroup people

**Treatment**	**SUCRA of OS**			
	PD-L1（+)	PD-L1（-)	MVI/EHS	MVI/EHS Absent
ICI+anti-VEGF Ab	**0.812**	0.489	0.56	0.636
Lenvatinib	-	-	0.31	0.263
Tremelimumab+durvalumab	0.563	0.746	0.65	**0.864**
Durvalumab	0.49	0.327	0.36	0.665
Nivolumab	0.479	**0.768**	0.817	0.341
HAIC-FO	-	-	**0.99**	0.606
Linifanib	-	-	0.007	0.615
Brivanib	-	-	0.189	0.185
Tislelizumab	-	-	0.593	0.656
ICI+anti-VEGF TKIs	-	-	0.703	0.303
Sorafenib	0.161	0.17	0.238	0.366

Abbreviations: **ICIs**, immune checkpoint inhibitors; **VEGF Ab**, vascular endothelial growth factor antibody; **TKIs**, tyrosine kinase inhibaitors; **OS**, overall survival; **PFS**, progression free survival; **ORR**, objective response rate; **AEs**, adverse events of grade 3 or higher; **TRSAEs**, treatment related serious adverse events; **MVI**, macrovascular invasion; **EHS**, extrahepatic metastasis; **TACE**, transcatheter arterial chemoembolization.

Bayesian ranking profiles of comparable treatments on efficacy for patients with advanced hepatocellular carcinoma.

Tables indicate the probability of each comparable treatment being ranked from first to last on overall survival, progression free survival, objective response rate, AEs and subgroup populations. The SUCRA value represents the likelihood of ranking probabilities. In terms of efficacy, the higher the SUCRA, the higher the likelihood that the regimen will cause better efficacy. In terms of security, the higher the SUCRA value, the higher the likelihood that the regimen will cause adverse events.

eFigure 1. Risk of bias graph for included studies

A. Risk of bias graph: review authors' judgements about each risk of bias item presented as percentages across all included studies

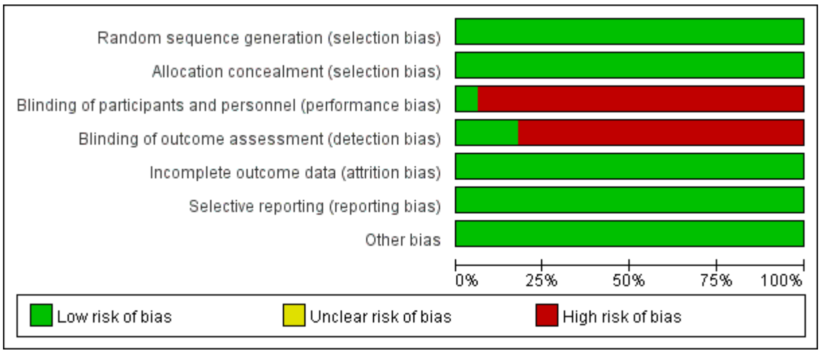

B. Risk of bias summary: review authors' judgements about each risk of bias item for each included study.

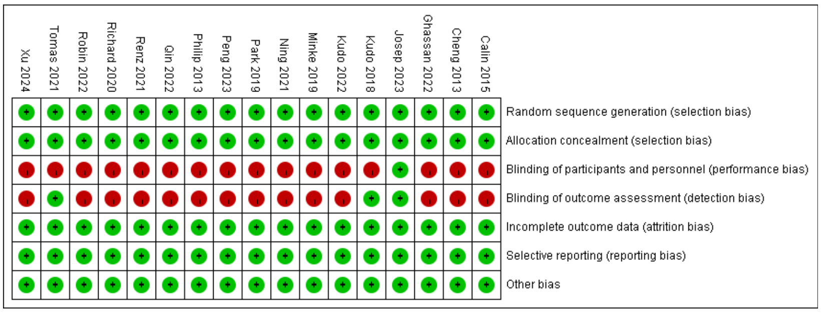

eFigure 2. Network diagrams of comparisons on different outcomes of treatments in patients with HCC.

A.

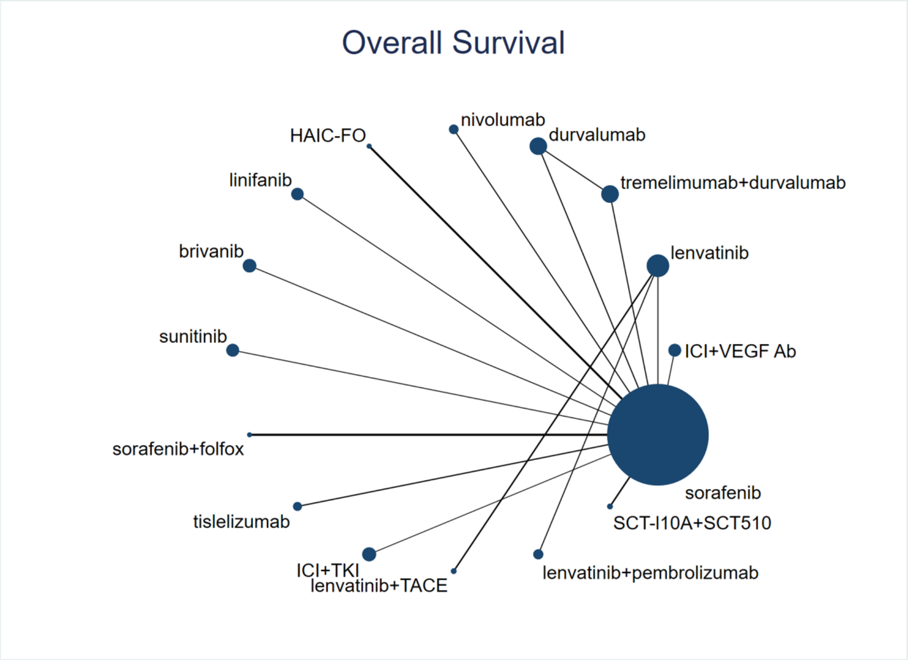

B.

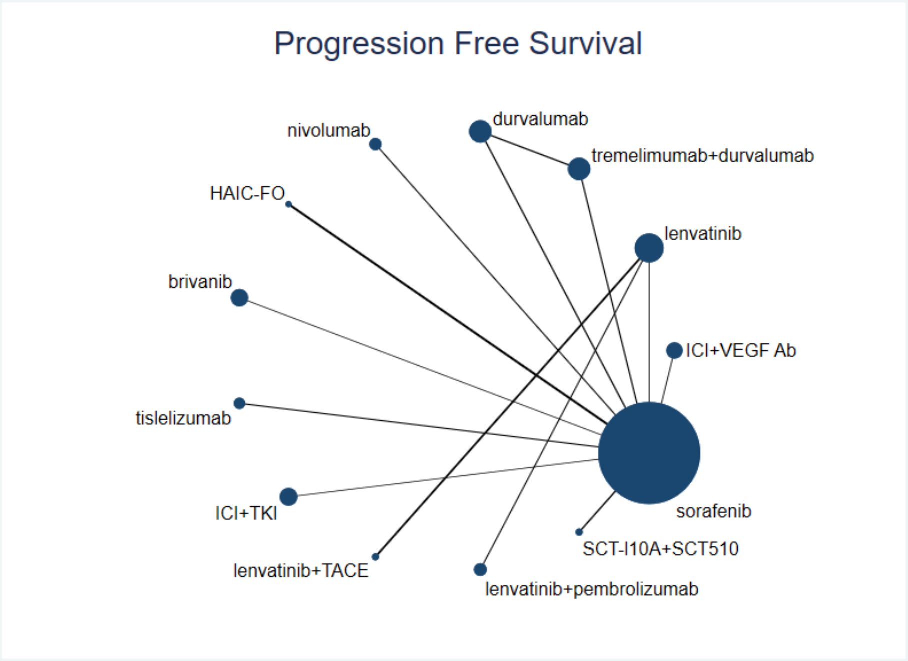

C.

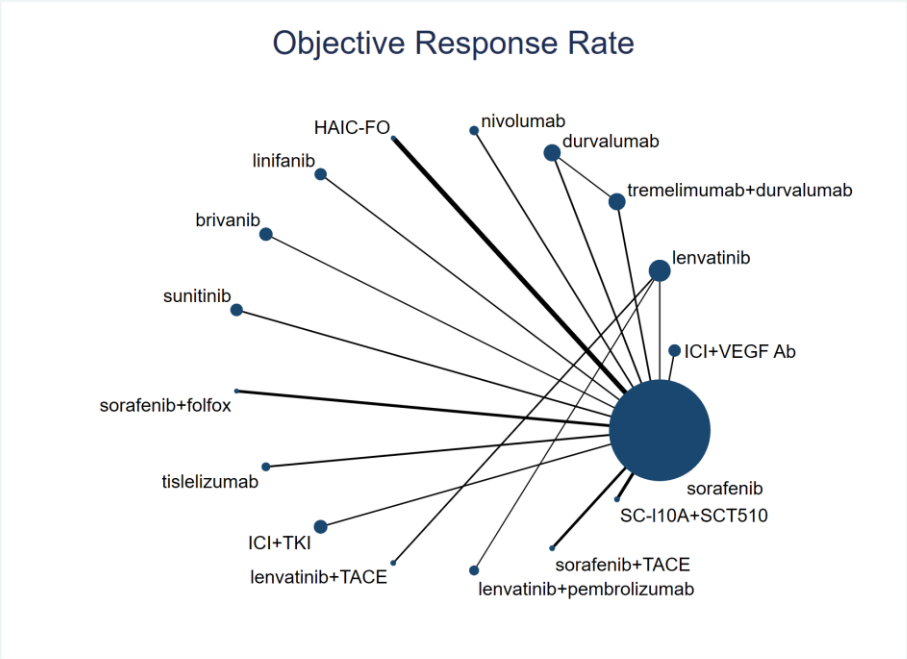
Objective Response Rate
HAIC-FO
nivolumab
durvalumab
linifanib
tremelimumab+durvalumab
brivanib
lenvatinib
sunitinib
ICI+VEGF Ab
sorafenib+folfox
tislelizumab
sorafenib
SC-I10A+SCT510
ICI+TKI
lenvatinib+TACE
sorafenib+TACE
lenvatinib+pembrolizumab

D.

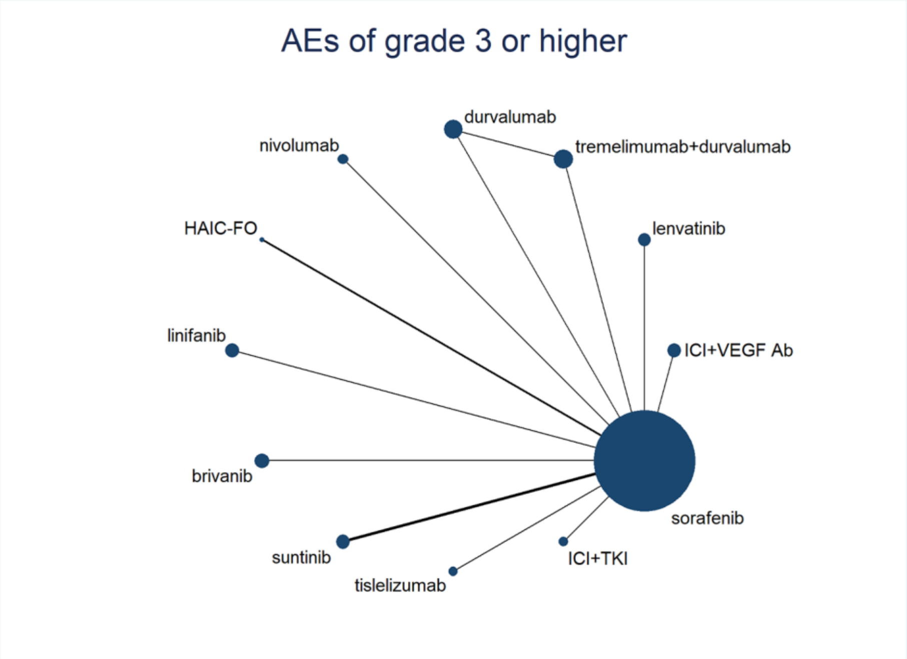
AEs of grade 3 or higher
durvalumab
nivolumab
tremelimumab+durvalumab
HAIC-FO
lenvatinib
linifanib
ICI+VEGF Ab
brivanib
sorafenib
suntinib
ICI+TKI
tislelizumab

E .

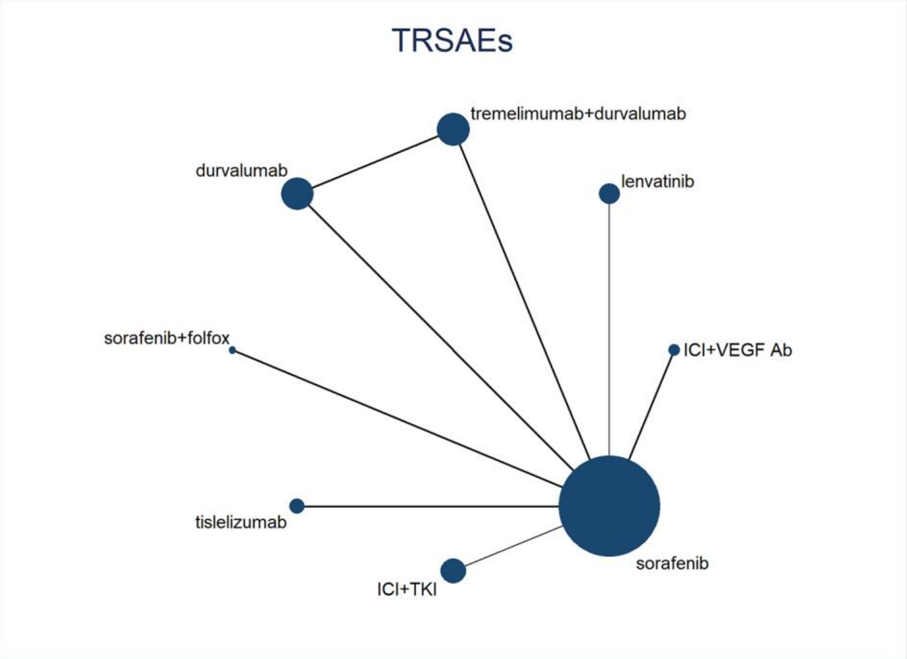

Abbreviations: ICIs, immune checkpoint inhibitors; VEGF Ab, vascular endothelial growth factor antibody; TKIs, tyrosine kinase inhibitors; AEs, adverse events; TRSAEs, treatment related serious adverse events.

Network diagrams of comparisons on different outcomes of treatments in different groups of patients with advanced hepatocellular carcinoma, the node size is proportional to the total number of patients receiving a treatment, the width of lines is proportional to the number of trials comparing the connected treatments.

(A) comparisons on overall survival in patients with advanced hepatocellular carcinoma
(B) comparisons on progression free survival in patients with advanced hepatocellular carcinoma
(C) comparisons on objective response rate in patients with advanced hepatocellular carcinoma
(D) comparisons on adverse events of grade 3 or higher in patients with advanced hepatocellular carcinoma
(E) comparisons on treatment related serious adverse events in patients with advanced hepatocellular carcinoma

eFigure 3. Network diagrams of comparisons on OS of treatments in different subgroups of patients with HCC.

A.

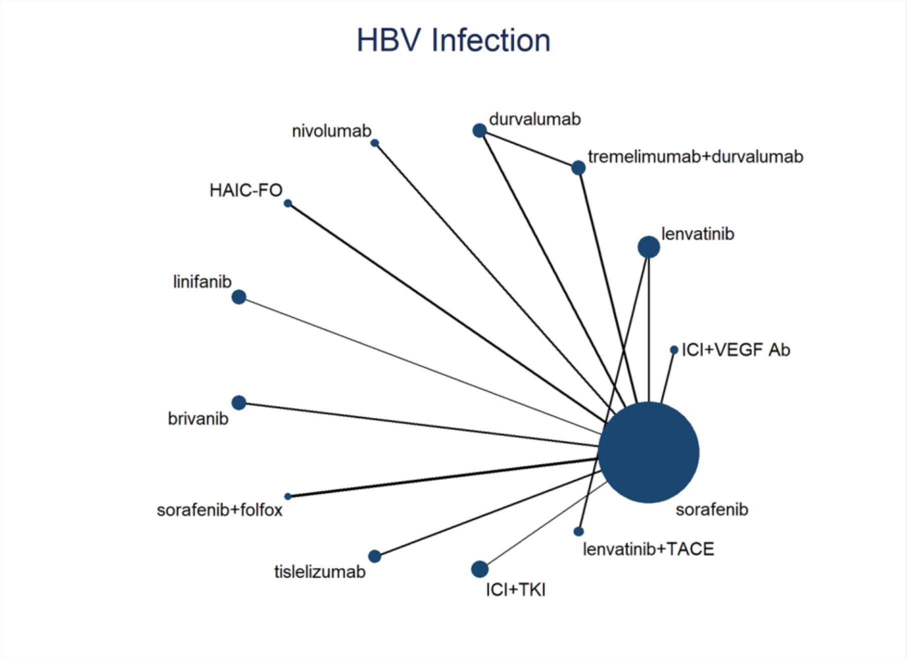

B.

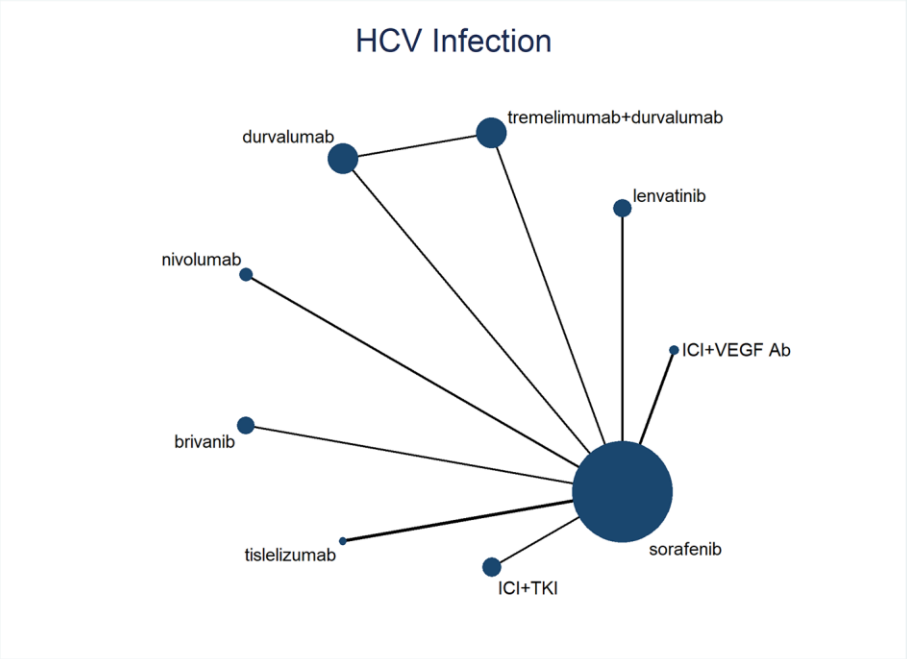

C.

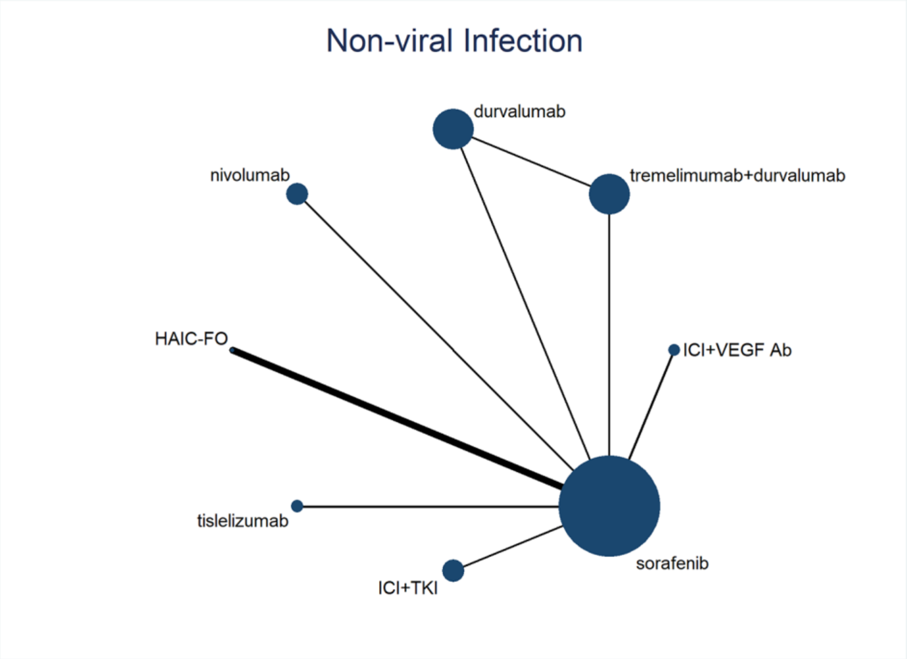
Non-viral Infection
durvalumab
nivolumab
tremelimumab+durvalumab
HAIC-FO
ICI+VEGF Ab
tislelizumab
ICI+TKI
sorafenib

D.

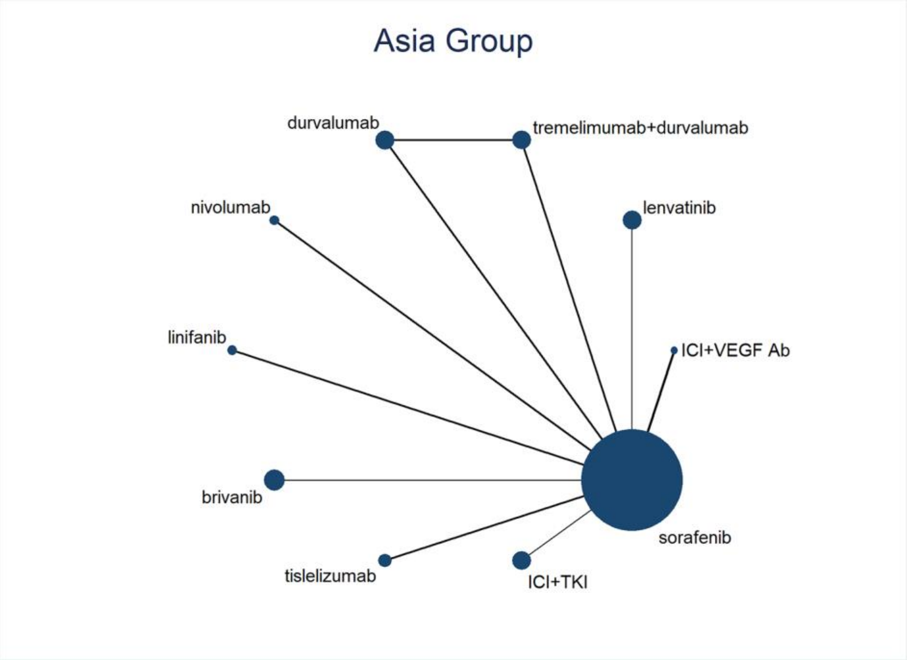
Asia Group
durvalumab
tremelimumab+durvalumab
nivolumab
lenvatinib
linifanib
ICI+VEGF Ab
brivanib
sorafenib
tislelizumab
ICI+TKI

E.

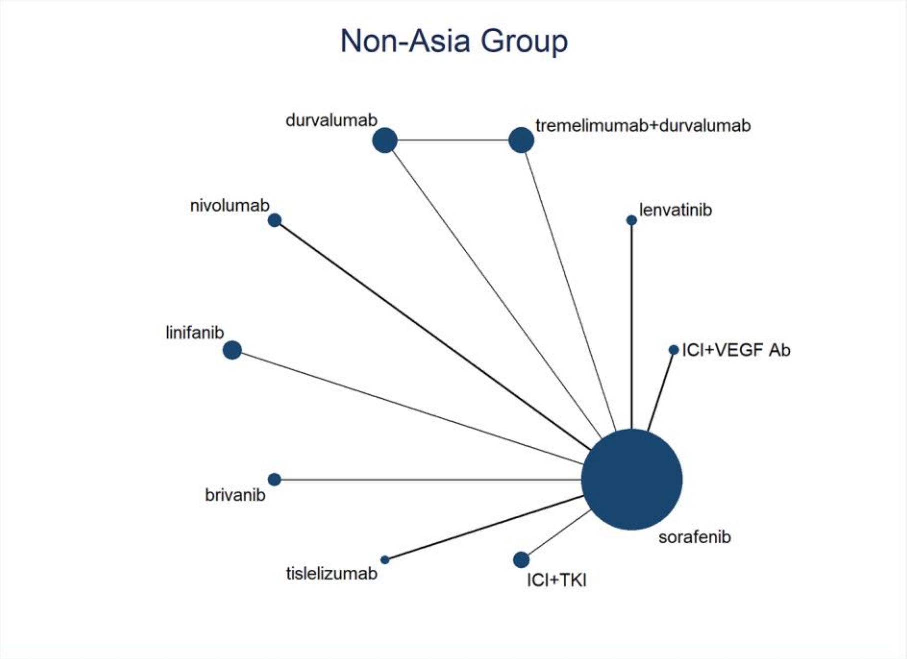
Non-Asia Group
durvalumab
tremelimumab+durvalumab
nivolumab
lenvatinib
linifanib
ICI+VEGF Ab
brivanib
sorafenib
tislelizumab
ICI+TKI

F.

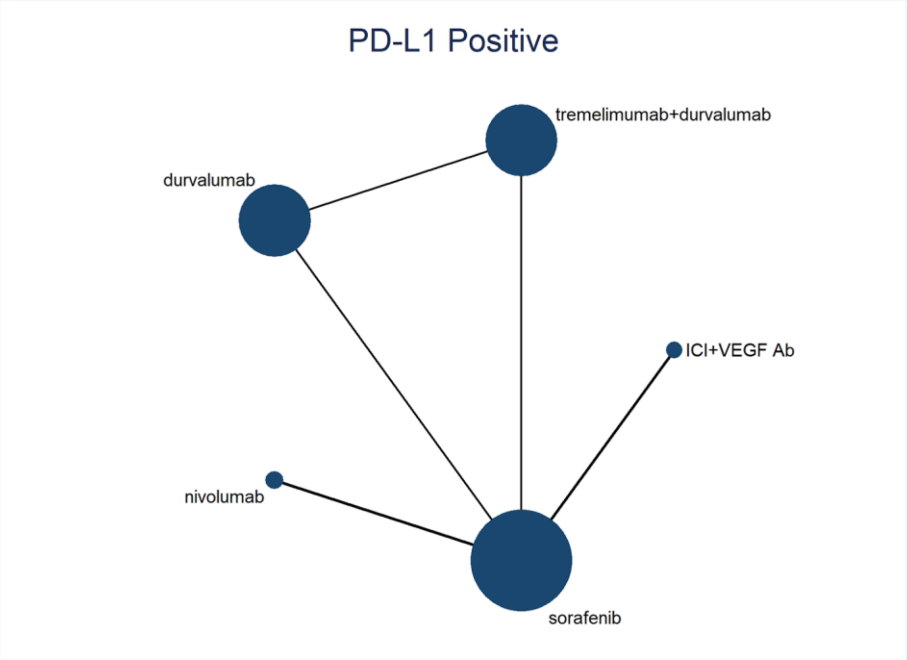
PD-L1 Positive
tremelimumab+durvalumab
durvalumab
ICI+VEGF Ab
nivolumab
sorafenib

G.

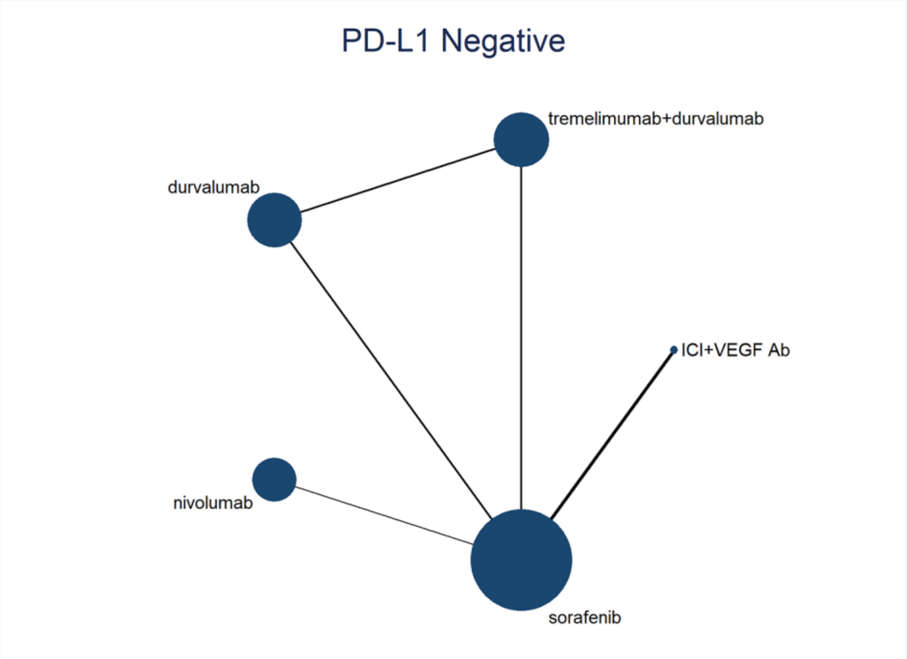
PD-L1 Negative
tremelimumab+durvalumab
durvalumab
ICI+VEGF Ab
nivolumab
sorafenib

H.

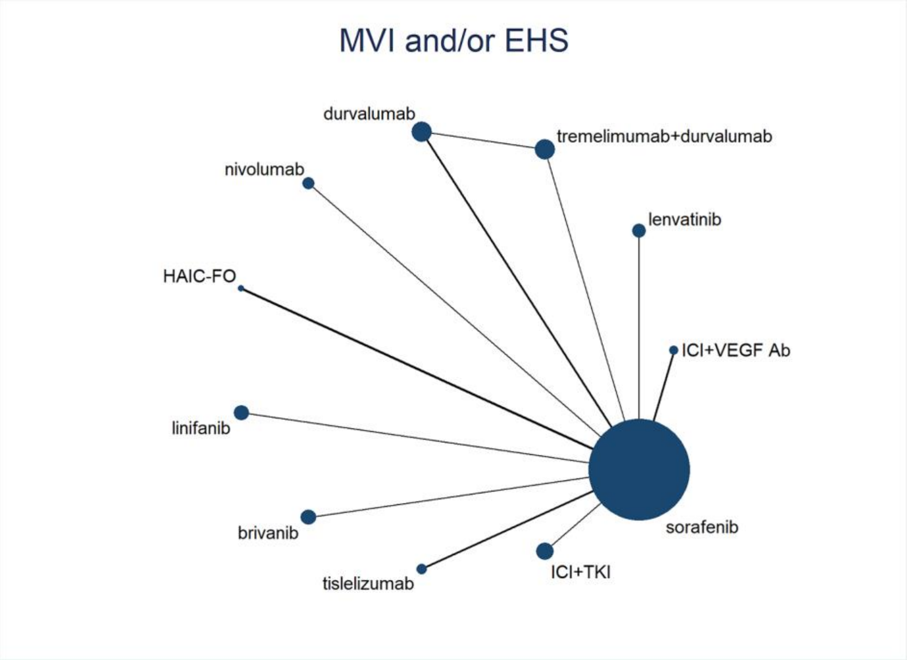
MVI and/or EHS
durvalumab
tremelimumab+durvalumab
nivolumab
lenvatinib
HAIC-FO
ICI+VEGF Ab
linifanib
brivanib
sorafenib
tislelizumab
ICI+TKI

I.

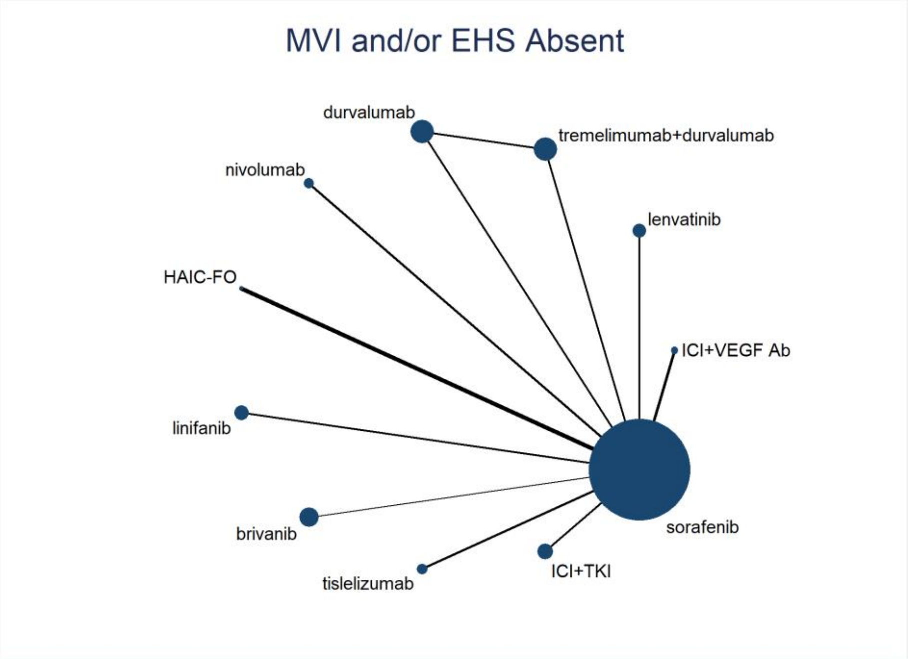

Abbreviations: ICIs, immune checkpoint inhibitors; VEGF Ab, vascular endothelial growth factor antibody; TKIs, tyrosine kinase inhibitors; MVI, macrovascular invasion; EHS, extrahepatic metastasis.

Network diagrams of comparisons on different outcomes of treatments in different subgroups of patients with advanced hepatocellular carcinoma, the node size is proportional to the total number of patients receiving a treatment, the width of lines is proportional to the number of trials. Comparing the connected treatments include

(A) comparisons on HBV infection in patients with advanced hepatocellular carcinoma
(B) comparisons on HCV infection in patients with advanced hepatocellular carcinoma
(C) comparisons on Nonviral infection in patients with advanced hepatocellular carcinoma
(D) comparisons on Asia group in patients with advanced hepatocellular carcinoma
(E) comparisons on Non Asia group in patients with advanced hepatocellular carcinoma
(F) comparisons on PD-L1 positive group in patients with advanced hepatocellular carcinoma
(G) comparisons on PD-L1 negative group in patients with advanced hepatocellular carcinoma
(H) comparisons on MVI/EHS group in patients with advanced hepatocellular carcinoma
(I) comparisons on MVI/EHS Absent group in patients with advanced hepatocellular carcinoma

eFigure 4. League table showing treatment-related serious adverse events

**TRSAEs**	**0.991**							
	ICI+TKI	**0.583**						
	**2.28 (1.19,4.35)**	ICI+VEFGAb	**0.712**					
	**1.88 (1.08,3.27)**	0.83 (0.44,1.55)	lenvainib	**0.783**				
	1.67 (0.93,3.03)	0.73 (0.38,1.43)	0.89 (0.50,1.58)	tremelimumab+ durvalumab	**0.168**			
	**3.96 (2.08,7.53)**	1.74 (0.85,3.54)	**2.10 (1.12,3.95)**	**2.36 (1.51,3.69)**	durvalumab	**0.163**		
	**4.00 (2.06,7.80)**	1.76 (0.84,3.66)	**2.13 (1.11,4.09)**	**2.39 (1.20,4.75)**	1.01 (0.49,2.10)	sorafenib+folfo x	**0.373**	
	**3.03 (1.61,5.71)**	1.33 (0.66,2.69)	1.61 (0.87,2.99)	1.81 (0.94,3.48)	0.77 (0.38,1.54)	0.76 (0.37,1.56)	tielelizumab	**0.227**
	**3.59 (2.40,5.37)**	1.57 (0.95,2.61)	**1.91 (1.30,2.79)**	**2.14 (1.39,3.31)**	0.91 (0.55,1.50)	0.90 (0.53,1.52)	1.18 (0.73,1.93)	sorafenib

Abbreviations: ICI, immune checkpoint inhibitor; VEGF Ab, vascular endothelial growth factor antibody; anti-VEGF TKIs, tyrosine kinase inhibitors; TRSAEs, treatment related serious adverse events.

Pooled hazard ratios (95% credible intervals) for TRSAEs. Each cell contains odds ratios (95% credible intervals) comparing the column-defining treatment to the row-defining treatment. Comparisons should be read from left to right. Odds ratios less than 1 favors column-defining treatment. Significant results are shown in bold.

eFigure 5. Pooled estimates of subgroup analyses

A. HBV Infection

HBV Infection

**0.982**												
sorafenib+folfox	**0.878**											
0.57 (0.21,1.52)	HAIC-FO	**0.695**										
0.37 (0.14,0.99)	0.65 (0.27,1.55)	lenvatinib+TACE	**0.765**									
0.42 (0.16,1.11)	0.73 (0.31,1.73)	1.13 (0.48,2.69)	tremelimumab+durvalumab	**0.658**								
**0.34 (0.15,0.77)**	0.59 (0.29,1.18)	0.91 (0.45,1.83)	0.80 (0.40,1.60)	ICI+TKI	**0.508**							
**0.28 (0.11,0.71)**	0.49 (0.21,1.11)	0.75 (0.33,1.72)	0.66 (0.29,1.51)	0.82 (0.43,1.57)	nivolumab	**0.461**						
**0.26 (0.10,0.71)**	0.46 (0.19,1.12)	0.71 (0.29,1.73)	0.63 (0.36,1.11)	0.78 (0.38,1.60)	0.95 (0.41,2.21)	durvalumab	**0.447**					
**0.25 (0.10,0.66)**	0.45 (0.19,1.03)	0.69 (0.30,1.60)	0.61 (0.27,1.40)	0.76 (0.40,1.46)	0.92 (0.42,2.04)	0.97 (0.41,2.26)	ICI+VEGF A	**0.32**				
**0.22 (0.09,0.53)**	**0.38 (0.18,0.81)**	0.59 (0.28,1.26)	0.52 (0.25,1.10)	0.65 (0.37,1.12)	0.79 (0.39,1.60)	0.83 (0.38,1.79)	0.85 (0.42,1.74)	tislelizumab	**0.202**			
**0.19 (0.08,0.45)**	**0.33 (0.16,0.69)**	**0.51 (0.32,0.82)**	**0.45 (0.22,0.94)**	**0.56 (0.33,0.95)**	0.68 (0.34,1.36)	0.72 (0.34,1.53)	0.74 (0.37,1.49)	0.87 (0.48,1.59)	lenvatinib	**0.178**		
**0.18 (0.08,0.43)**	**0.32 (0.16,0.66)**	**0.50 (0.24,1.02)**	**0.44 (0.21,0.89)**	**0.54 (0.33,0.90)**	0.66 (0.34,1.29)	0.69 (0.33,1.45)	0.72 (0.36,1.41)	0.84 (0.47,1.50)	0.97 (0.55,1.69)	linifanib	**0.253**	
**0.20 (0.08,0.48)**	**0.35 (0.17,0.74)**	**0.54 (0.26,1.15)**	**0.48 (0.23,1.00)**	**0.60 (0.35,1.02)**	0.73 (0.36,1.46)	0.76 (0.36,1.63)	0.79 (0.39,1.59)	0.92 (0.50,1.70)	1.06 (0.59,1.91)	1.10 (0.62,1.93)	brivanib	**0.155**
**0.18 (0.09,0.40)**	**0.32 (0.17,0.60)**	**0.50 (0.27,0.92)**	**0.44 (0.24,0.80)**	**0.55 (0.39,0.76)**	0.66 (0.38,1.16)	0.70 (0.37,1.31)	0.72 (0.41,1.26)	0.84 (0.54,1.31)	0.97 (0.64,1.46)	1.00 (0.69,1.46)	0.91 (0.60,1.39)	sorafenib

B. HCV Infection

HCV Infection

**0.875**								
ICI+VEGF Ab	**0.438**							
0.43 (0.16,1.16)	ICI+TKI	**0.186**						
**0.31 (0.11,0.84)**	0.71 (0.33,1.51)	tremelumumab +durvalumab	**0.125**					
**0.28 (0.10,0.78)**	0.65 (0.30,1.39)	0.92 (0.52,1.62)	durvalumab	**0.713**				
0.67 (0.23,1.94)	1.55 (0.68,3.54)	2.19 (0.93,5.17)	**2.39 (1.01,5.65)**	nivolumab	**0.851**			
0.97 (0.27,3.44)	2.24 (0.76,6.55)	**3.16 (1.05,9.49)**	**3.44 (1.14,10.37)**	1.44 (0.46,4.54)	tislelizumab	**0.293**		
0.36 (0.13,1.00)	0.83 (0.39,1.79)	1.18 (0.53,2.62)	1.28 (0.57,2.87)	0.54 (0.23,1.28)	0.37 (0.12,1.12)	lenvatinib	**0.698**	
0.64 (0.24,1.75)	1.48 (0.70,3.13)	2.10 (0.96,4.58)	2.28 (1.04,5.01)	0.96 (0.41,2.23)	0.66 (0.22,1.98)	1.78 (0.81,3.94)	brivanib	**0.321**
**0.38 (0.16,0.88)**	0.87 (0.53,1.45)	1.23 (0.70,2.16)	1.34 (0.76,2.37)	0.56 (0.29,1.08)	0.39 (0.15,1.00)	1.05 (0.59,1.86)	0.59 (0.34,1.01)	sorafenib

C. Nonviral Infection

Non-Viral Infection

**0.964**							
HAIC-FO	**0.783**						
0.24 (0.03,1.72)	tremelimumab+ durvalumab	**0.193**					
**0.11 (0.02,0.81)**	**0.47 (0.24,0.92)**	TKI+ICI	**0.125**				
0.10 (0.01,0.74)	**0.41 (0.17,0.94)**	0.86 (0.38,1.93)	ICI+VEGF Ab	**0.614**			
0.19 (0.03,1.38)	0.80 (0.50,1.29)	1.69 (0.86,3.32)	1.98 (0.85,4.63)	durvalumab	**0.537**		
0.17 (0.02,1.22)	0.71 (0.36,1.42)	1.50 (0.78,2.89)	1.75 (0.76,4.04)	0.89 (0.44,1.78)	nivolumab	**0.517**	
0.17 (0.02,1.29)	0.70 (0.30,1.66)	1.48 (0.65,3.40)	1.73 (0.65,4.61)	0.88 (0.37,2.08)	0.99 (0.42,2.31)	tislelizumab	**0.267**
**0.12 (0.02,0.86)**	**0.53 (0.32,0.87)**	1.12 (0.72,1.74)	1.30 (0.66,2.58)	0.66 (0.40,1.09)	0.74 (0.46,1.20)	0.75 (0.37,1.52)	sorafenib

D. Asia Group

Aisa Group

**0.88**									
tremelimumab+ durvalumab	**0.4**								
**0.57 (0.35,0.93)**	durvalumab	**0.82**							
0.91 (0.45,1.83)	1.59 (0.77,3.25)	nivolumab	**0.58**						
0.69 (0.30,1.61)	1.21 (0.51,2.85)	0.76 (0.33,1.75)	ICI+VEGF Ab	**0.76**					
0.82 (0.45,1.49)	1.43 (0.77,2.65)	0.90 (0.50,1.61)	1.19 (0.56,2.50)	ICI+TKI	**0.43**				
0.58 (0.31,1.09)	1.02 (0.54,1.94)	0.64 (0.35,1.18)	0.85 (0.39,1.82)	0.71 (0.44,1.16)	lenvatinib	**0.13**			
**0.42 (0.21,0.84)**	0.74 (0.37,1.49)	**0.47 (0.24,0.91)**	0.61 (0.27,1.39)	**0.52 (0.29,0.91)**	0.72 (0.40,1.31)	linifanib	**0.18**		
**0.47 (0.25,0.87)**	0.82 (0.44,1.54)	**0.52 (0.28,0.94)**	0.68 (0.32,1.45)	**0.57 (0.36,0.92)**	0.80 (0.48,1.33)	1.11 (0.62,1.98)	brivanib	**0.5**	
0.63 (0.32,1.24)	1.10 (0.55,2.20)	0.69 (0.36,1.35)	0.91 (0.40,2.05)	0.77 (0.44,1.34)	1.07 (0.60,1.93)	1.49 (0.77,2.85)	1.34 (0.76,2.37)	tislelizumab	**0.29**
**0.52 (0.31,0.87)**	0.91 (0.54,1.54)	**0.57 (0.35,0.93)**	0.75 (0.39,1.47)	**0.64 (0.46,0.88)**	0.89 (0.62,1.29)	1.23 (0.77,1.97)	1.11 (0.79,1.57)	0.83 (0.53,1.31)	sorafenib

E. Non-Asia Group

Non-Asia Group

**0.66**									
ICI+VEGF Ab	**0.3**								
0.73 (0.40,1.36)	ICI+TKI	**0.66**							
0.98 (0.51,1.87)	1.34 (0.79,2.27)	tremelimumab+ durvaumab	**0.6**						
0.94 (0.49,1.80)	1.28 (0.75,2.19)	0.96 (0.65,1.42)	durvalumab	**0.64**					
0.97 (0.50,1.88)	1.33 (0.77,2.28)	0.99 (0.56,1.76)	1.03 (0.58,1.85)	nivolumab	**0.83**				
1.21 (0.58,2.54)	1.66 (0.87,3.14)	1.24 (0.64,2.41)	1.29 (0.66,2.53)	1.25 (0.63,2.47)	tislelizumab	**0.16**			
0.62 (0.31,1.25)	0.85 (0.47,1.53)	0.64 (0.34,1.18)	0.66 (0.36,1.23)	0.64 (0.34,1.20)	0.51 (0.25,1.05)	lenvatinib	**0.69**		
1.00 (0.55,1.82)	1.37 (0.86,2.18)	1.02 (0.62,1.69)	1.07 (0.64,1.78)	1.03 (0.61,1.74)	0.83 (0.44,1.54)	1.61 (0.91,2.83)	linifanib	**0.12**	
0.61 (0.32,1.16)	0.83 (0.49,1.41)	0.62 (0.36,1.09)	0.65 (0.37,1.14)	0.63 (0.35,1.12)	**0.50 (0.26,0.98)**	0.98 (0.53,1.81)	0.61 (0.37,1.01)	brivanib	**0.29**
0.74 (0.45,1.23)	1.01 (0.71,1.43)	0.76 (0.51,1.12)	0.79 (0.53,1.18)	0.76 (0.50,1.16)	0.61 (0.36,1.04)	1.19 (0.74,1.90)	0.74 (0.54,1.01)	1.21 (0.82,1.81)	sorafenib

F. PD-L1 positive

PD_L1 positive (Overall Survival)

**0.812**				
ICI+VEFGF Ab	**0.563**			
0.71 (0.27,1.84)	tremelimumab+ durvalumab	**0.49**		
0.67 (0.26,1.74)	0.94 (0.58,1.54)	durvalumab	**0.475**	
0.66 (0.22,2.01)	0.94 (0.37,2.33)	0.99 (0.40,2.47)	nivolumab	**0.161**
0.52 (0.23,1.18)	0.74 (0.45,1.23)	0.79 (0.48,1.30)	0.79 (0.37,1.70)	sorafenib

F. PD-L1 negative

PD-L1 Negative(Overall Survival)

**0.489**				
ICI+VEGFAb	**0.746**			
1.21 (0.42,3.51)	tremelimumab+ durvalumab	**0.327**		
0.89 (0.30,2.57)	0.73 (0.47,1.14)	durvalumab	**0.768**	
1.25 (0.45,3.46)	1.03 (0.58,1.83)	1.41 (0.78,2.54)	nivolumab	**0.17**
0.79 (0.31,2.07)	0.65 (0.41,1.03)	0.90 (0.56,1.44)	0.64 (0.45,1.03)	sorafenib

Abbreviations: ICI, immune checkpoint inhibitor; VEGF Ab, vascular endothelial growth factor antibody; TKIs, tyrosine kinase inhibitors.

(A) Pooled hazard ratios (95% credible intervals) for HBV Infection subgroup. (B) Pooled hazard ratios (95% credible intervals) for HCV Infection subgroup. (C) Pooled hazard ratios (95% credible intervals) for Nonviral Infection subgroup. (D) Pooled hazard ratios (95% credible intervals) for Asia subgroup. (E) Pooled hazard ratios (95% credible intervals) for Non-Asia subgroup. (F) Pooled hazard ratios (95% credible intervals) for PD-L1 positive subgroup. (G) Pooled hazard ratios (95% credible intervals) for PD-L1 negative subgroup. Comparisons should be read from left to right. Hazard ratios less than 1 favors column-defining treatment. Significant results are shown in bold.

eFigure 6. Forest plot of Frequentist network meta-analysis using random-effects model

A. Overall Survival

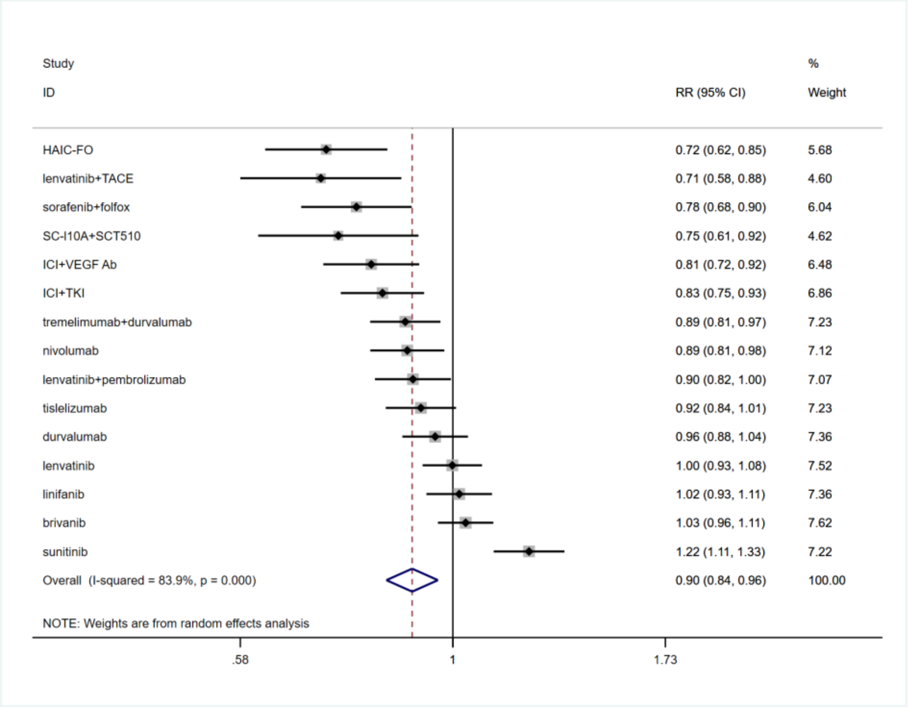

B. Progression Free Survival

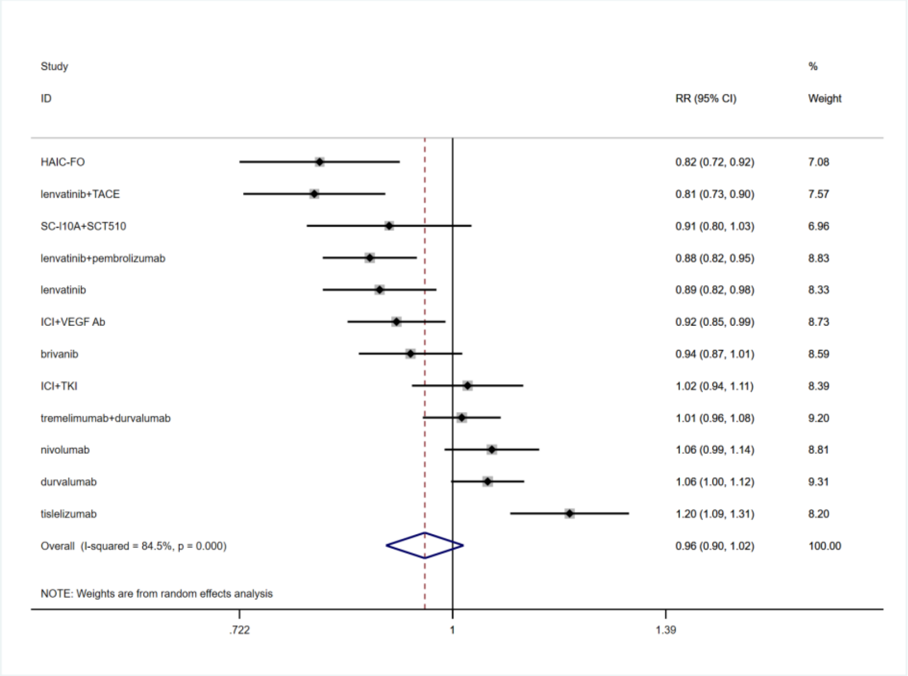

C.Objective Response Rate

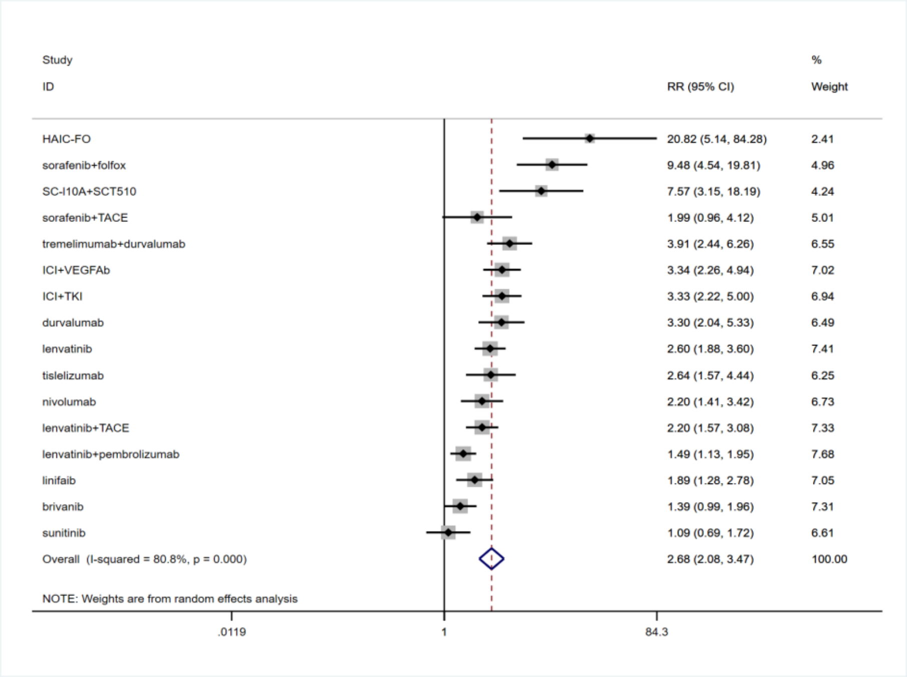

D. HBV Infection

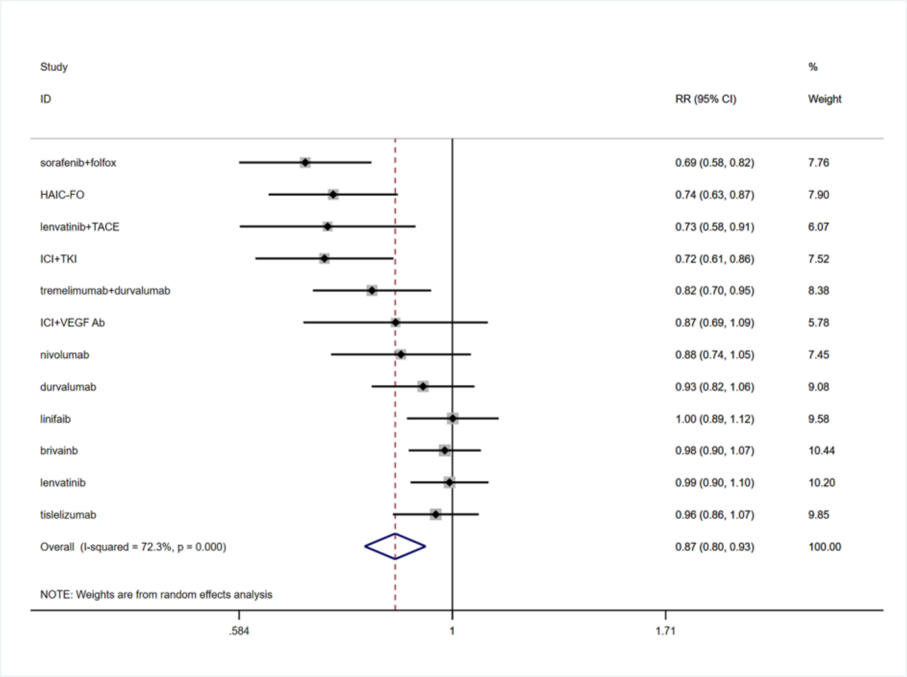

E. HCV Infection

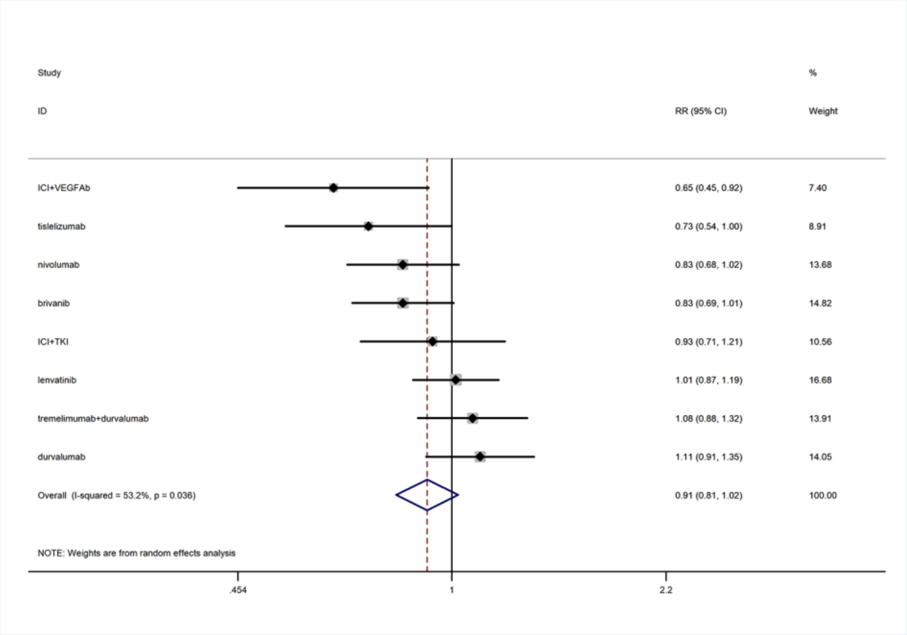

F. Nonviral Infection

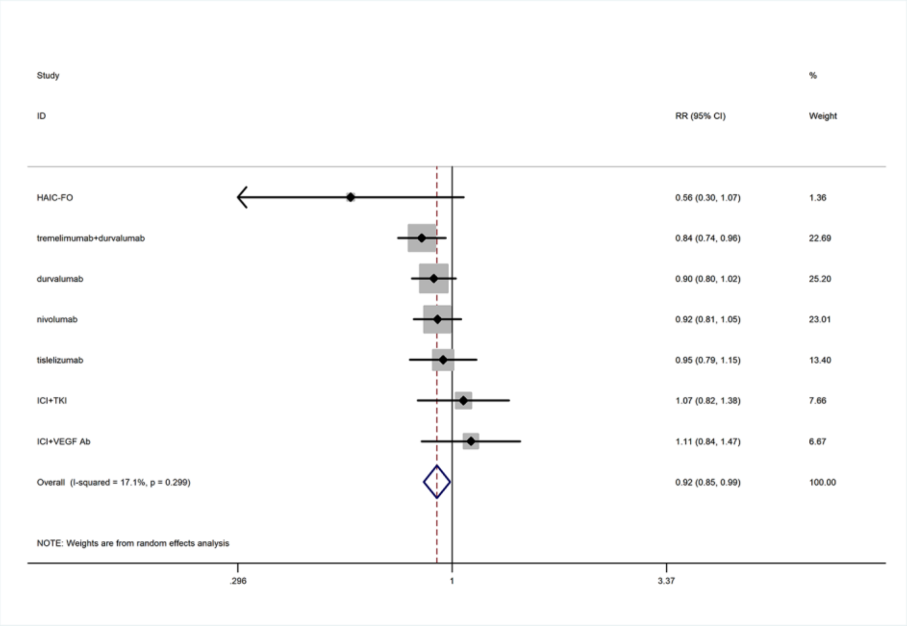

G. Asia group

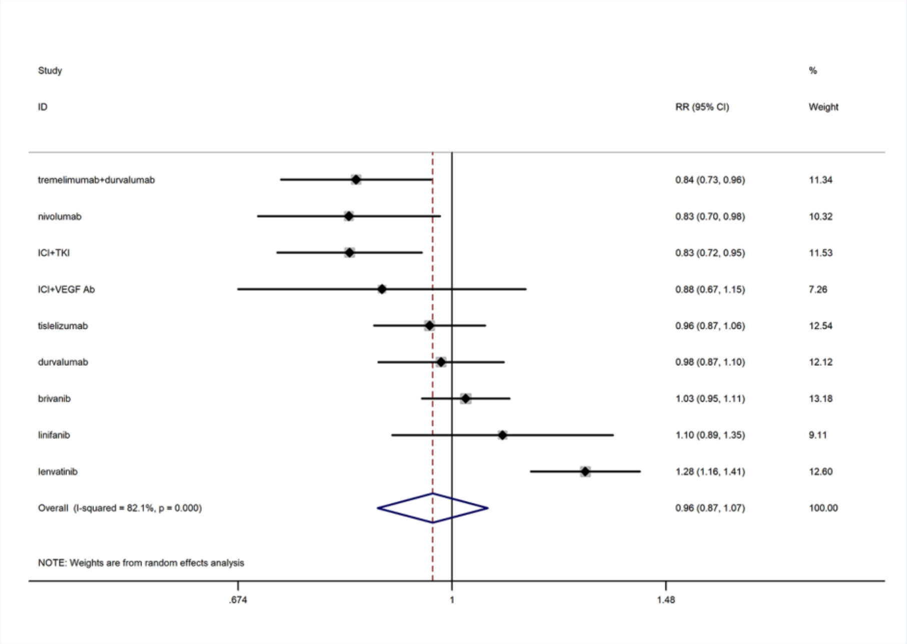

H. Non-Asia group

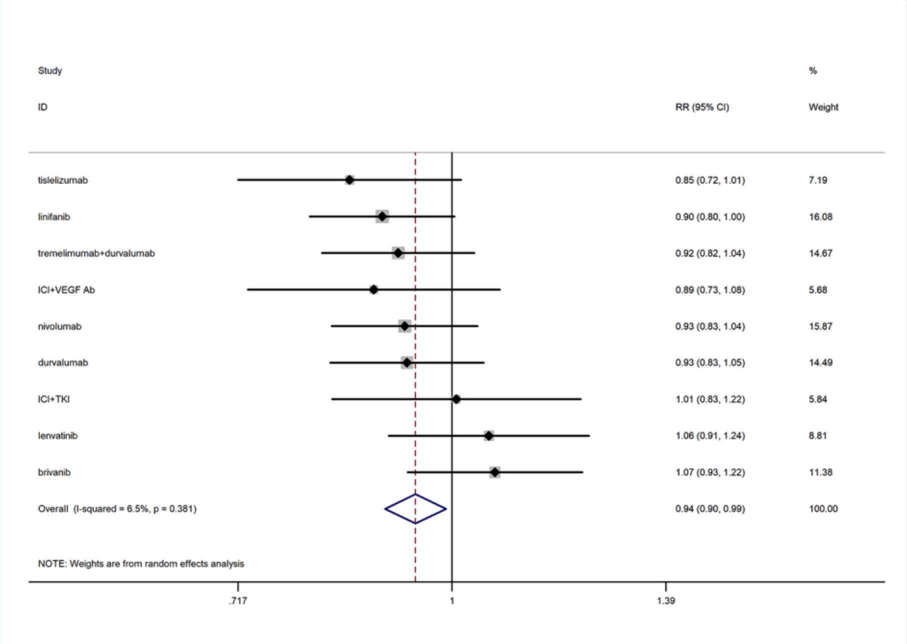

I. PD-L1 positive

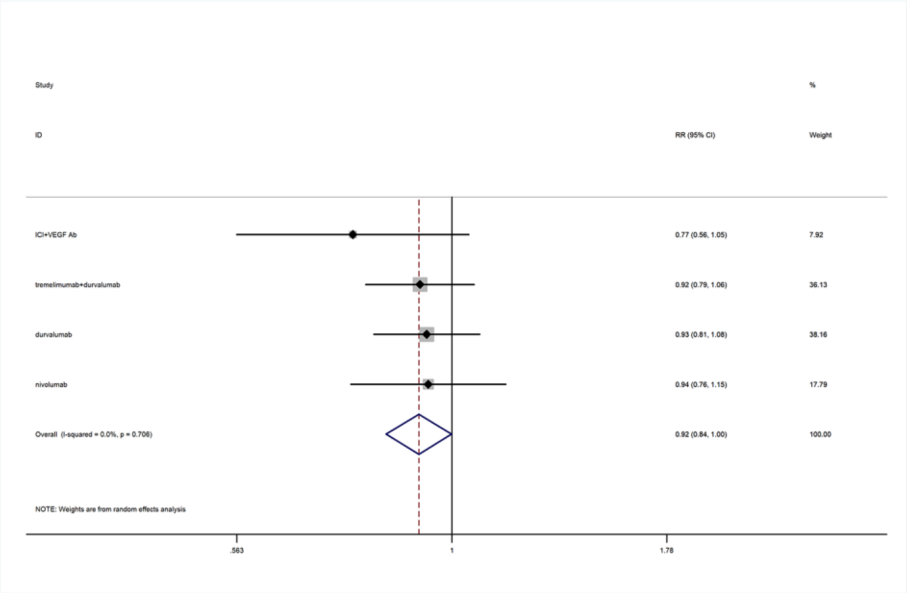

J. PD-L1 negative

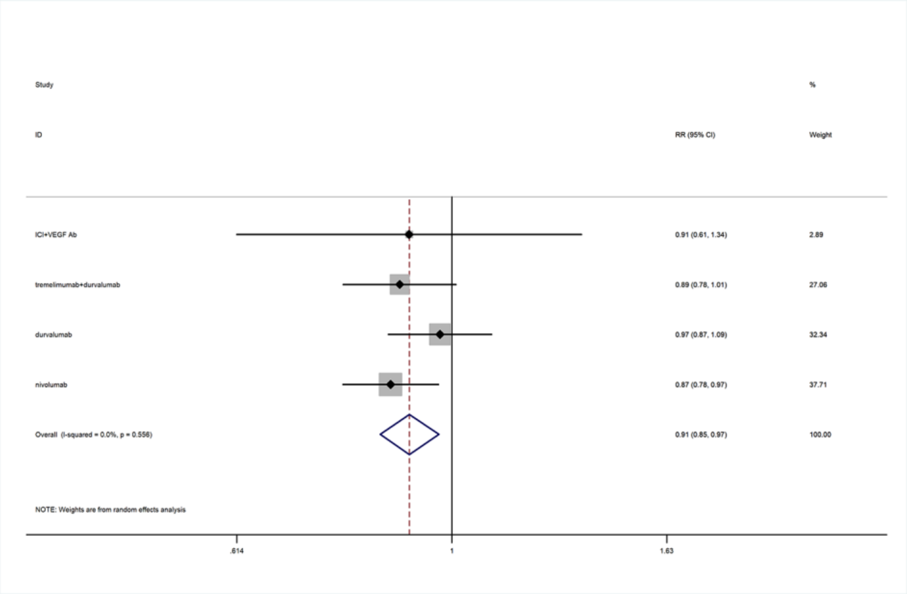

K. MVI and/or EHS

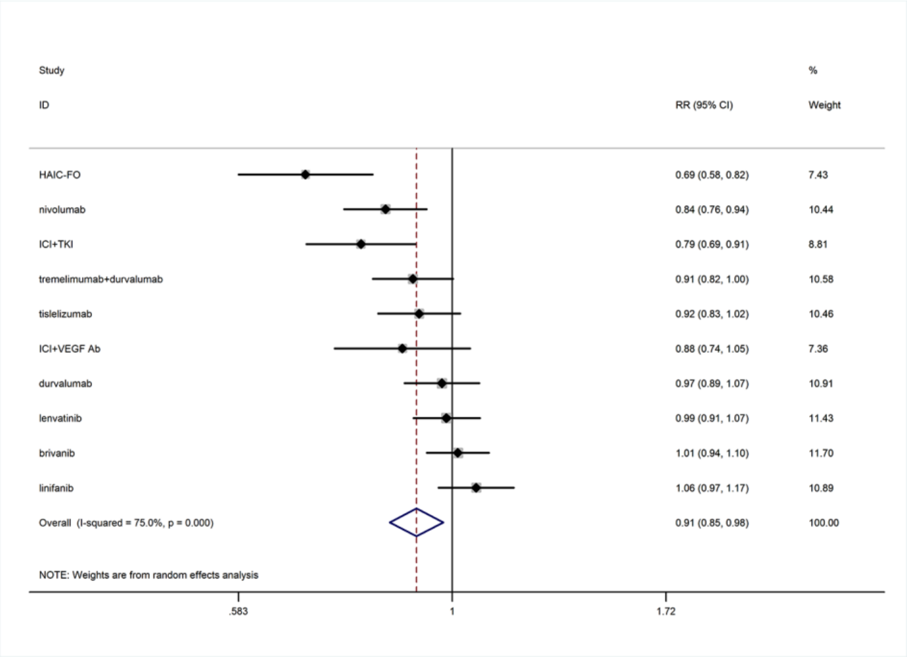

L. MVI and/or EHS Absent

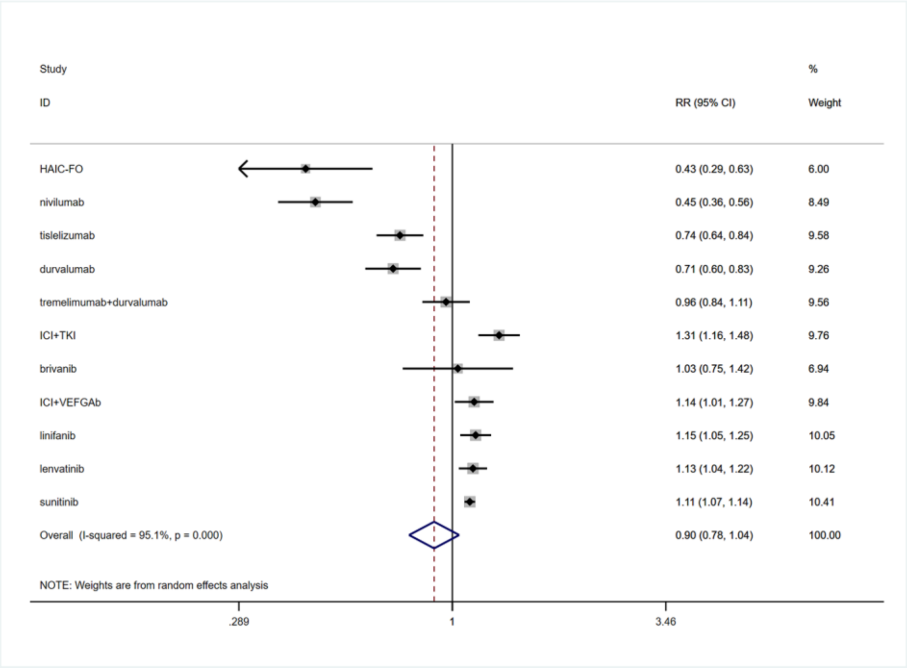

M. AEs of grade 3 or higher

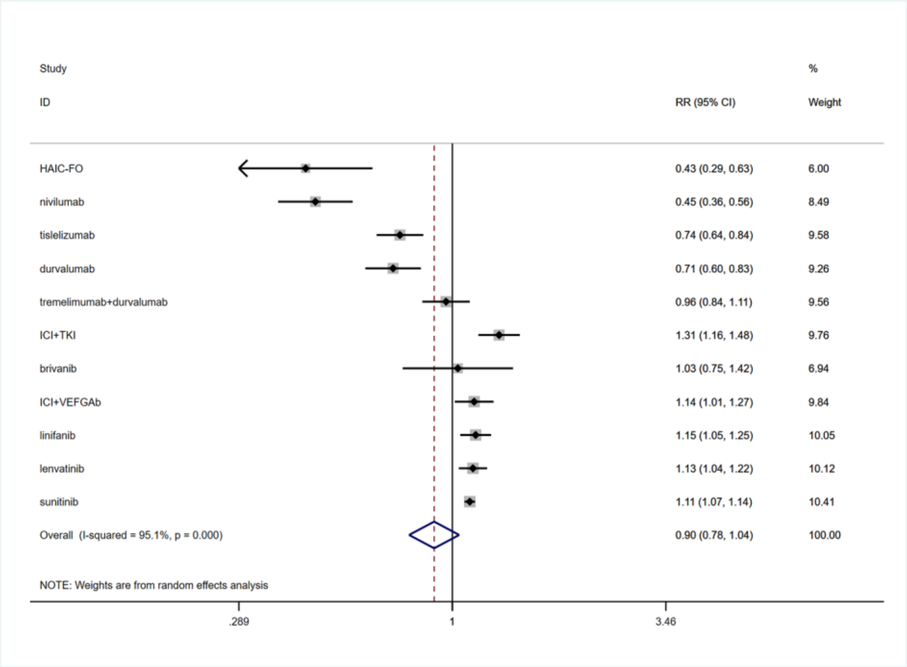

N. TRSAEs

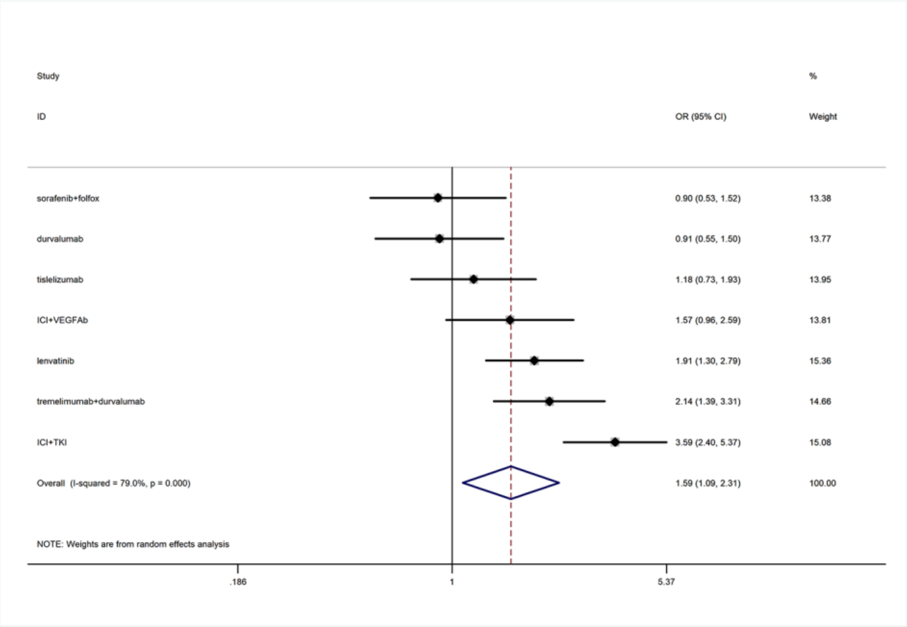

Abbreviations: ICI, immune checkpoint inhibitor; VEGF Ab, vascular endothelial growth factor antibody; TKIs, tyrosine kinase inhibitors; MVI, macrovascular invasion; EHS, extrahepatic metastasis; AEs, adverse events; TRSAEs, treatment related serious adverse events.

Forest plots depicting results of head-to-head comparisons according different outcomes in advanced hepatocellular. All controls were sorafenib. I^2 represents the amount of heterogeneity between studies. Heterogeneity was considered low, moderate,

or high for estimated I^2 values under 25%, between 25% and 50%, and over 50%, respectively.

eFigure 7. Analysis of sensitivity

A. Overall Survival

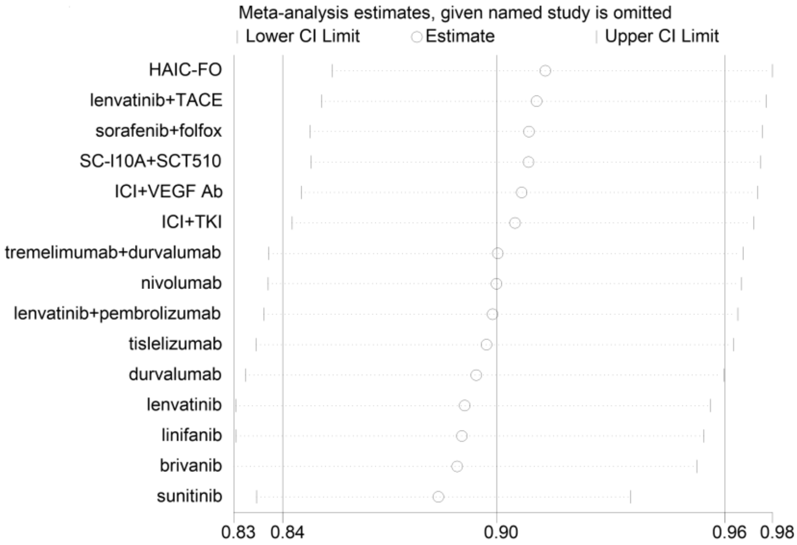

B. Progression Free Survival

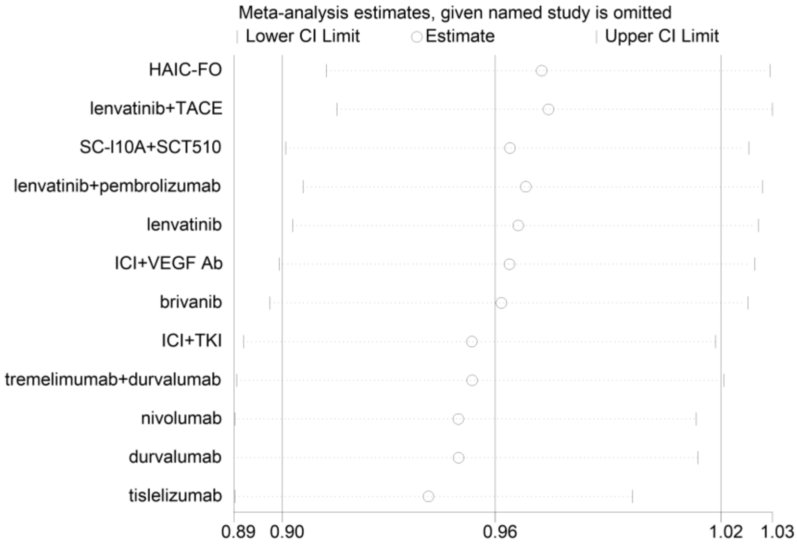

C. Objective response rate

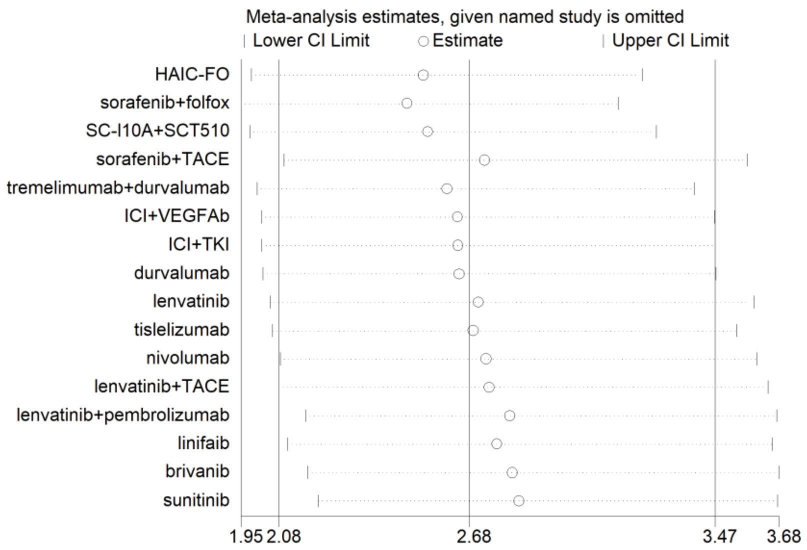

D. Asia Group

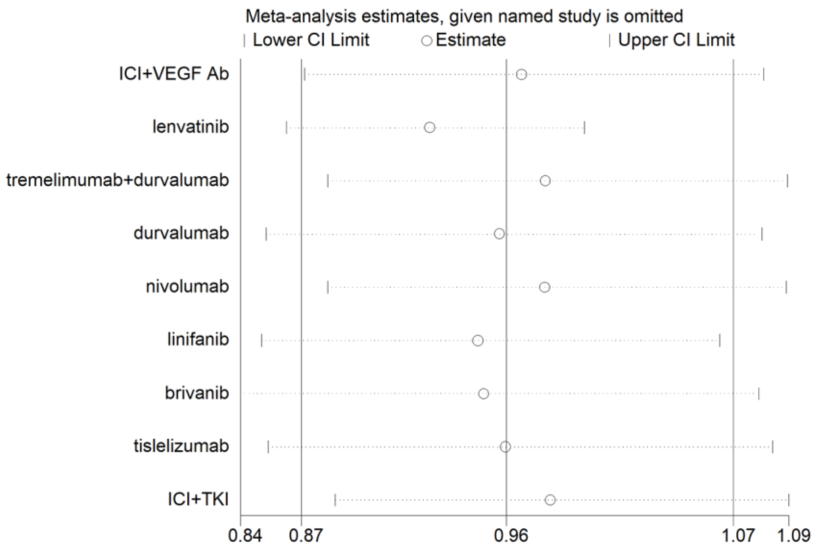

E. Non-Asia

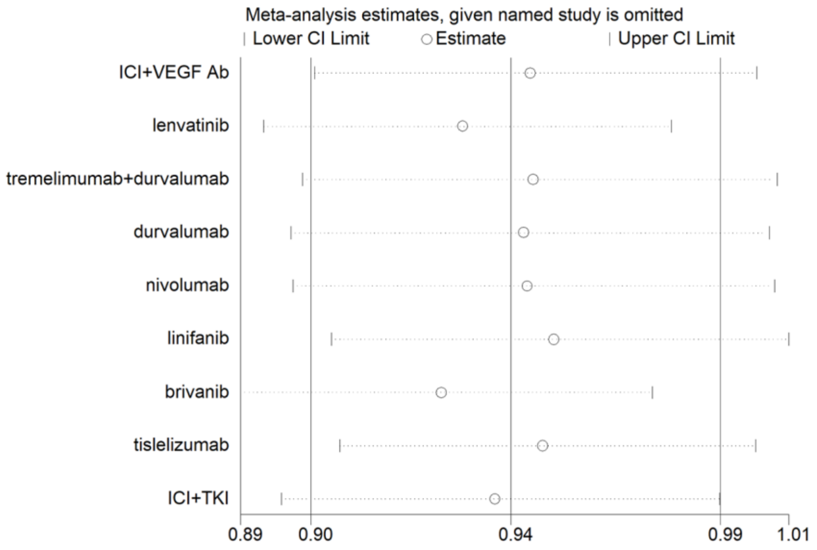

F. HBV-Infection

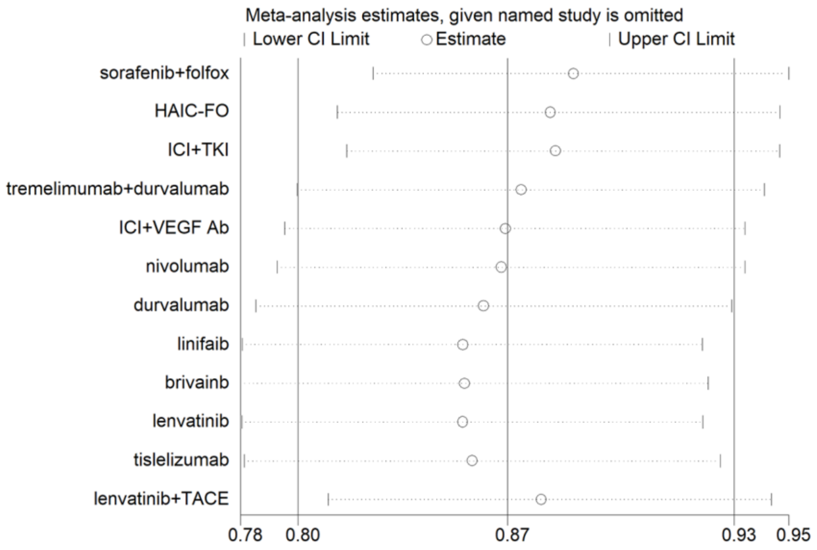

G. HCV Infection

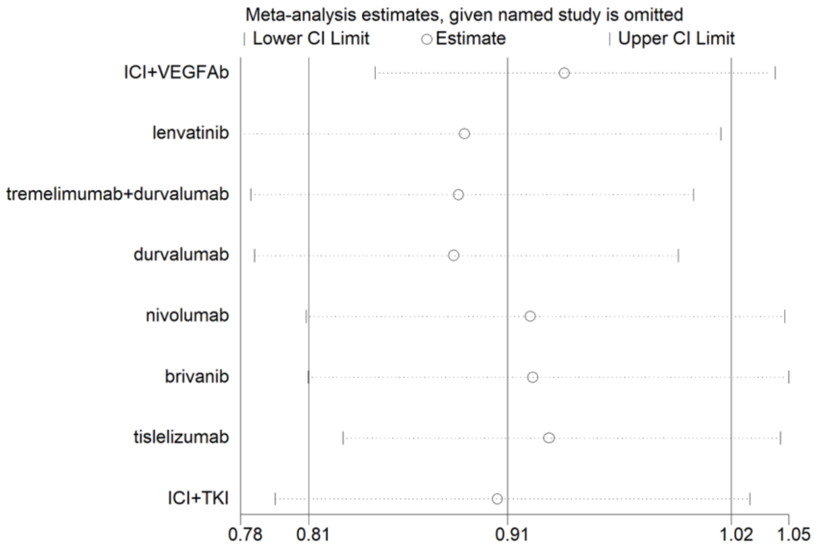

H. Non-Viral Infection

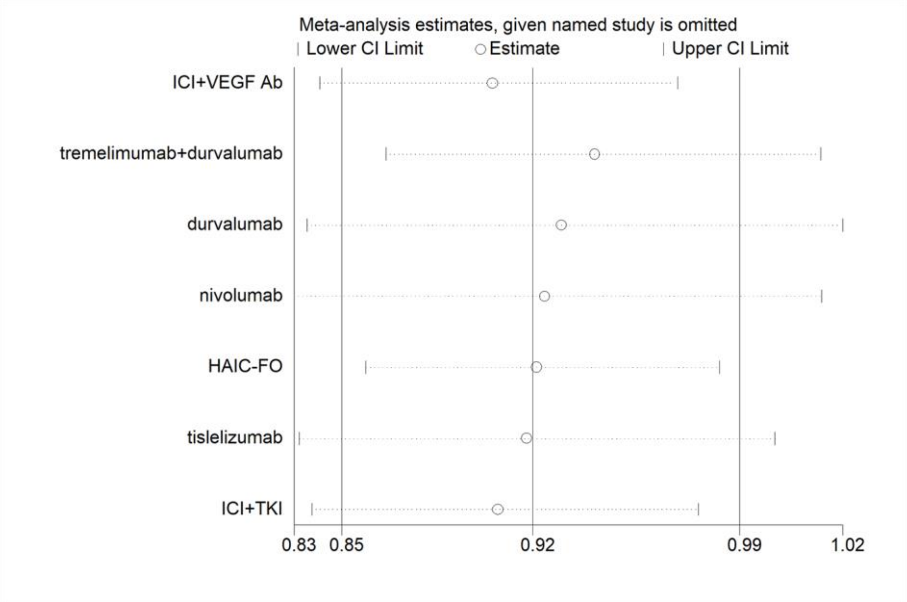

I. MVI and/or EHS

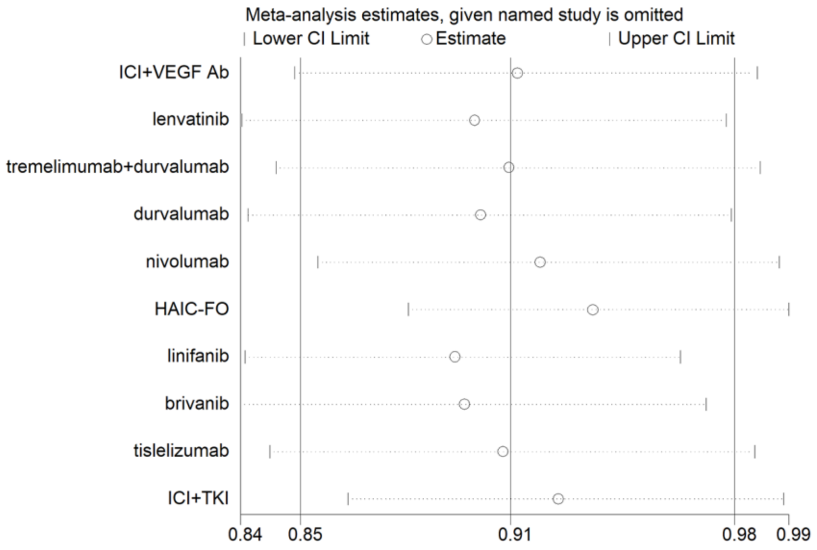

J. MVI and/or EHS Absent

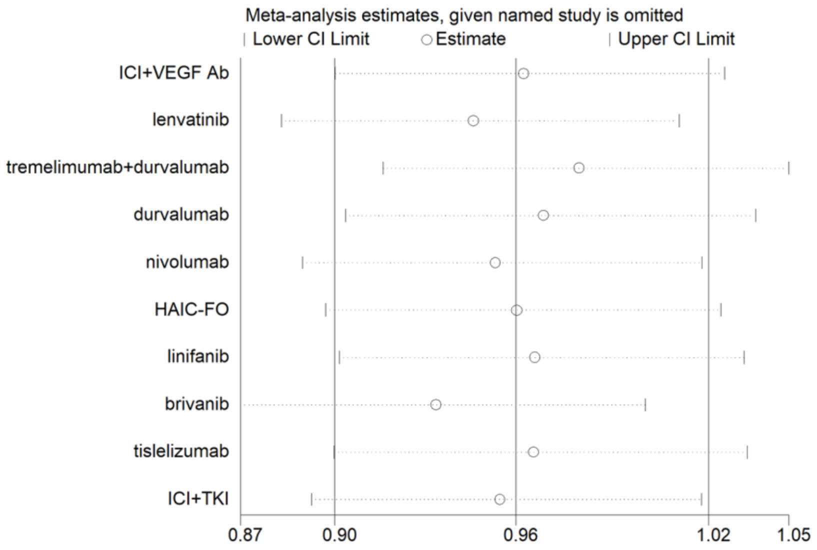

K. PD-L1 positive

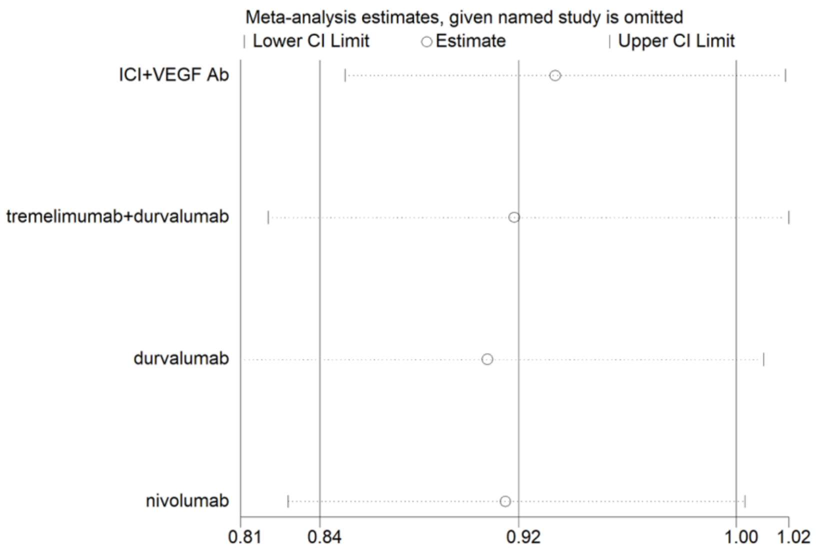

L. PD-L1 negative

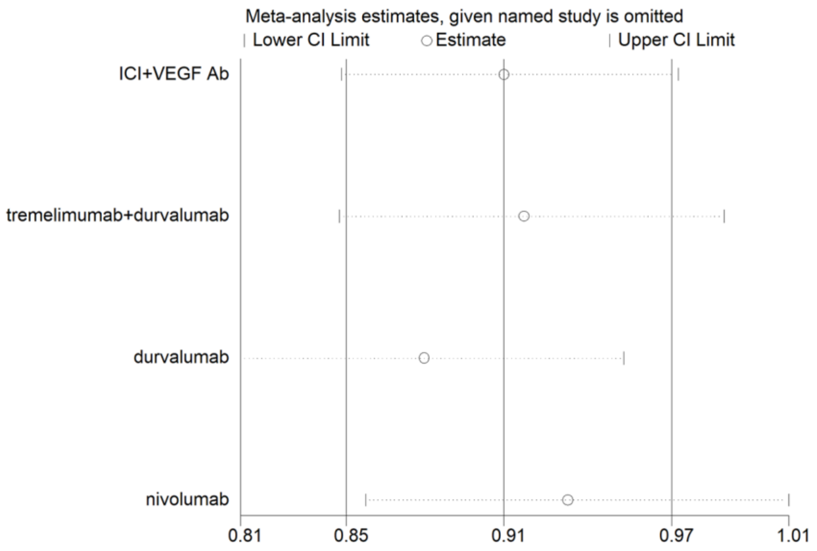

M. AEs of grade 3 or higher

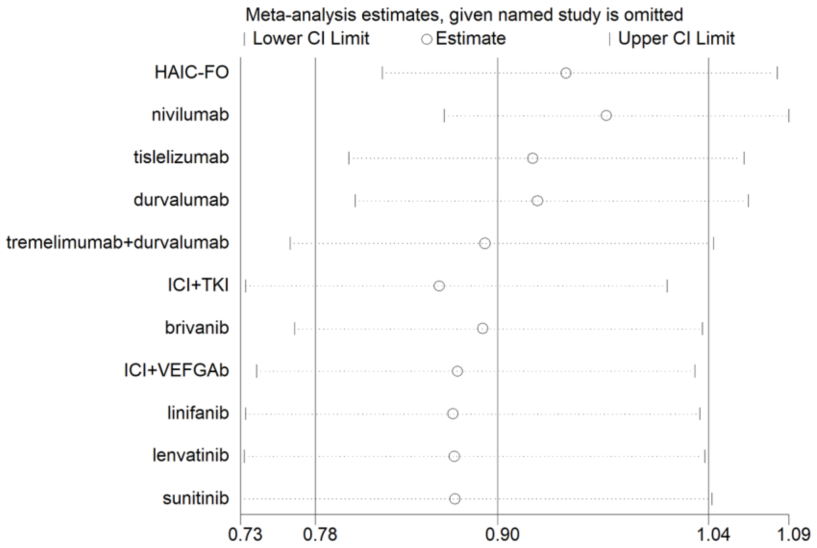

N. TRSAEs

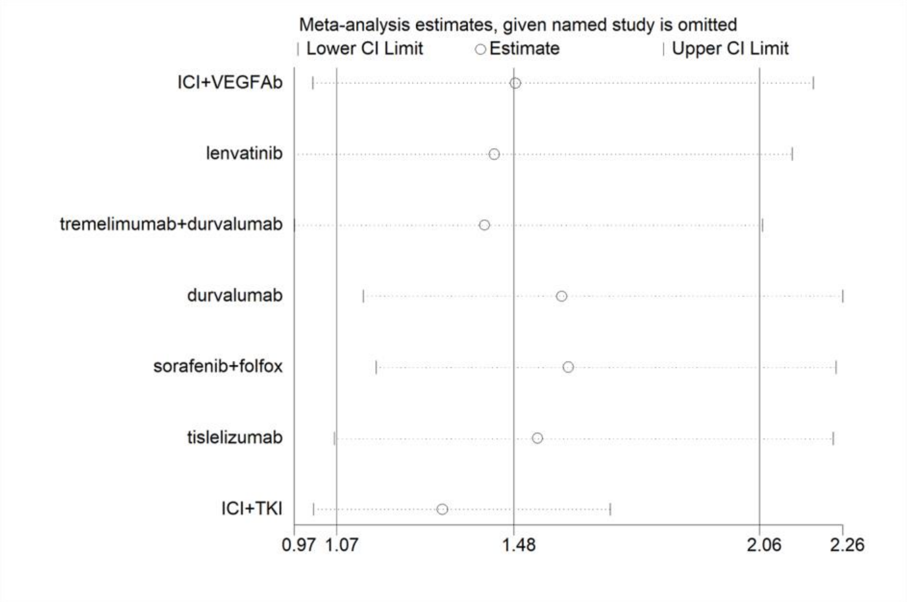

Abbreviations: ICI, immune checkpoint inhibitor; VEGF Ab, vascular endothelial growth factor antibody; TKIs, tyrosine kinase inhibitors; MVI, macrovascular invasion; EHS, extrahepatic metastasis; AEs, adverse events; TRSAEs, treatment related serious adverse events.

Sensitivity analysis was carried out by step elimination. If most studies were eliminated, the combined results of the remaining studies were not statistically significant (95% CI included 1), indicating that the results of the original meta-analysis were prone to significant changes due to the number of studies, and the model lacked robustness.

eFigure 8. Ranking curves displaying the probabilities of different regimens

A. Asia Group

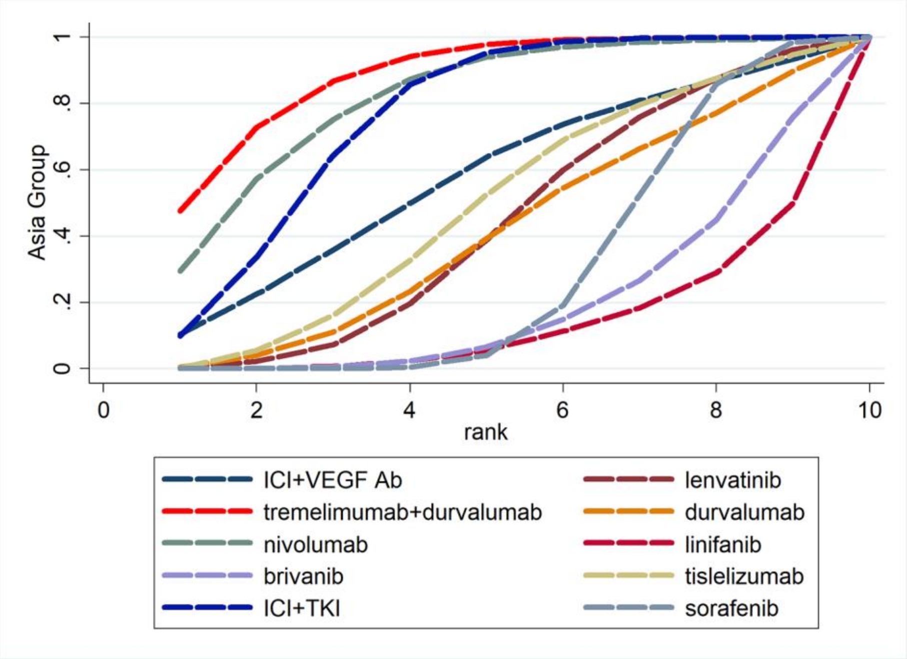

B. Non-Asia Group

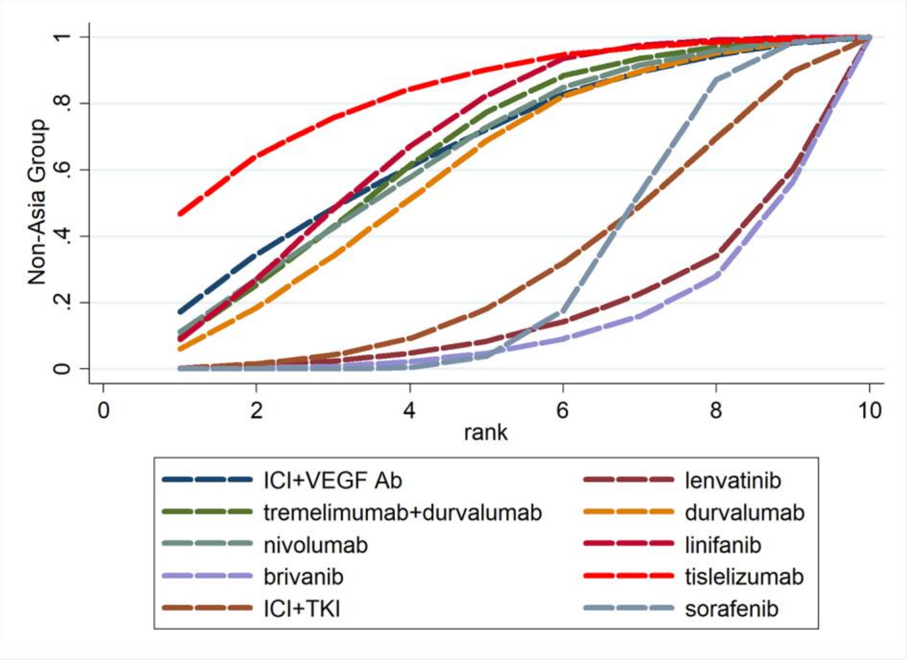

C. HBV infection

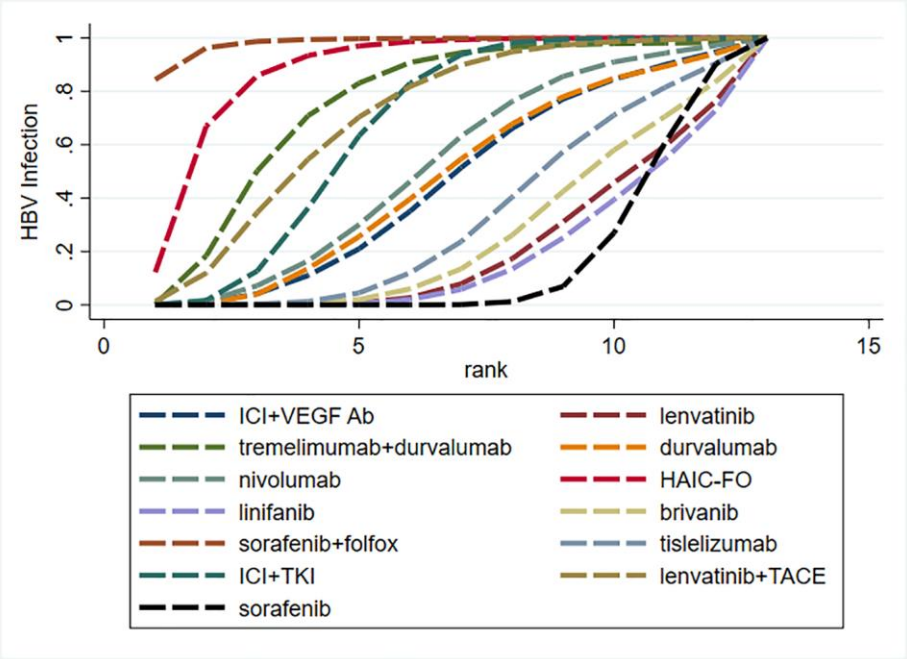

D. HCV-Infection

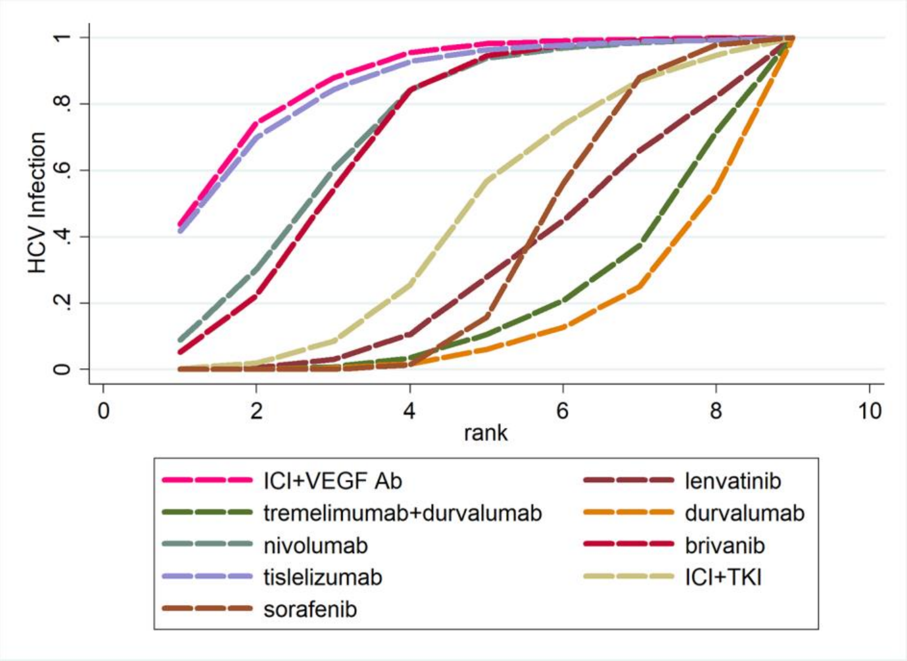

E. Non-Viral Infection

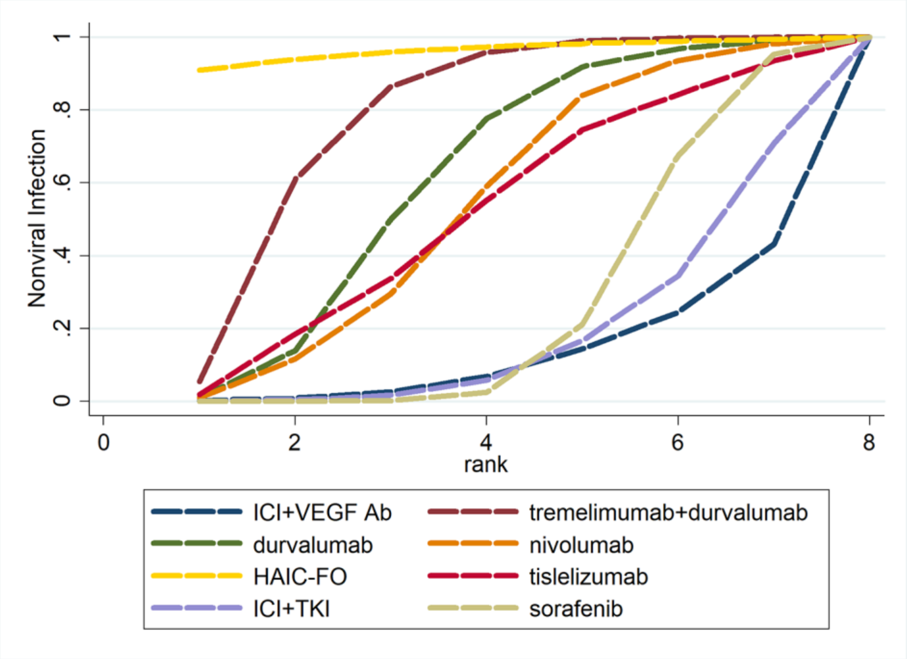

F. MVI and/or EHS

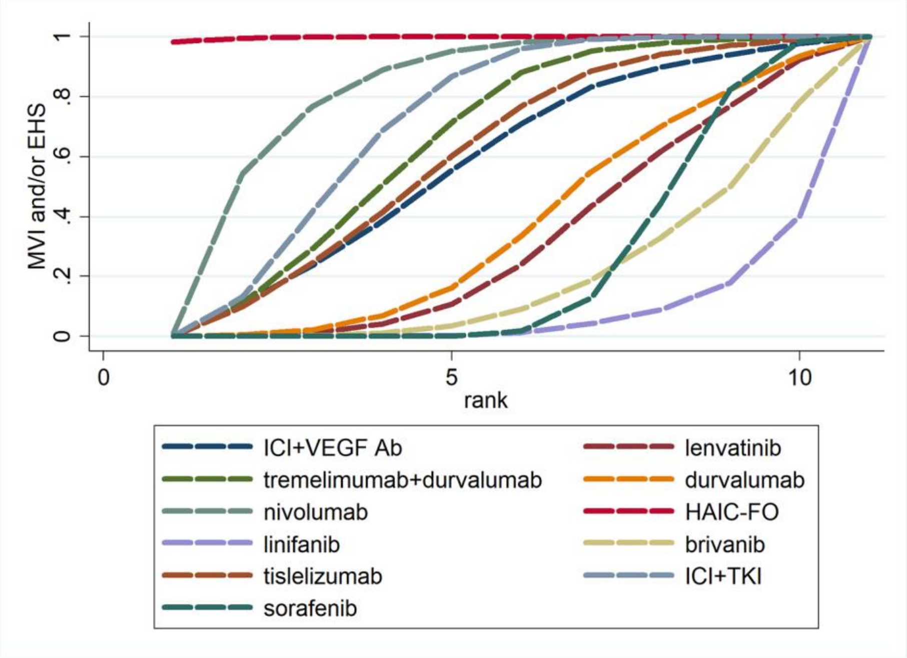

G. MVI and/or EHS Absent

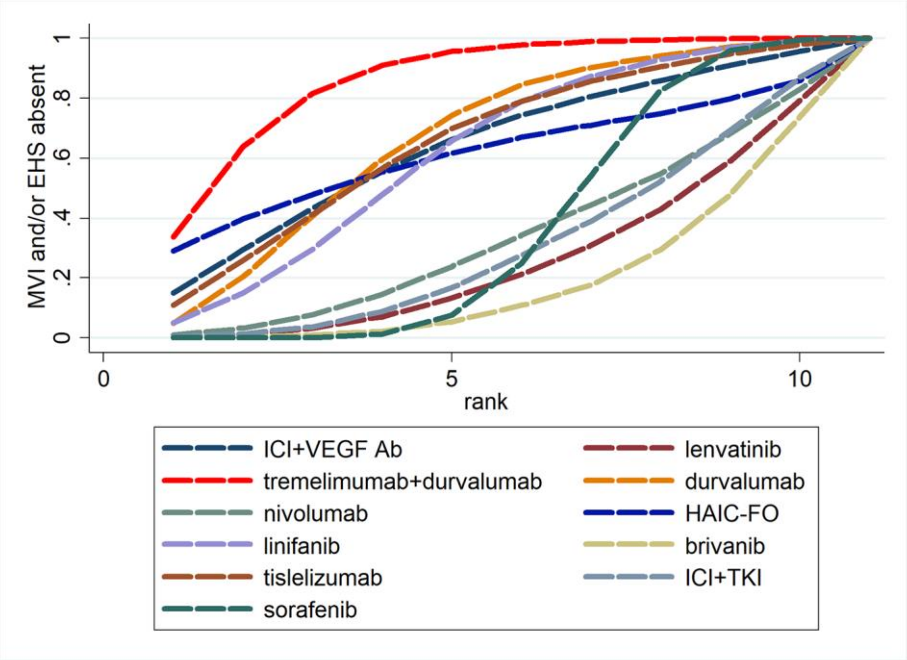

H. PD-L1 positive

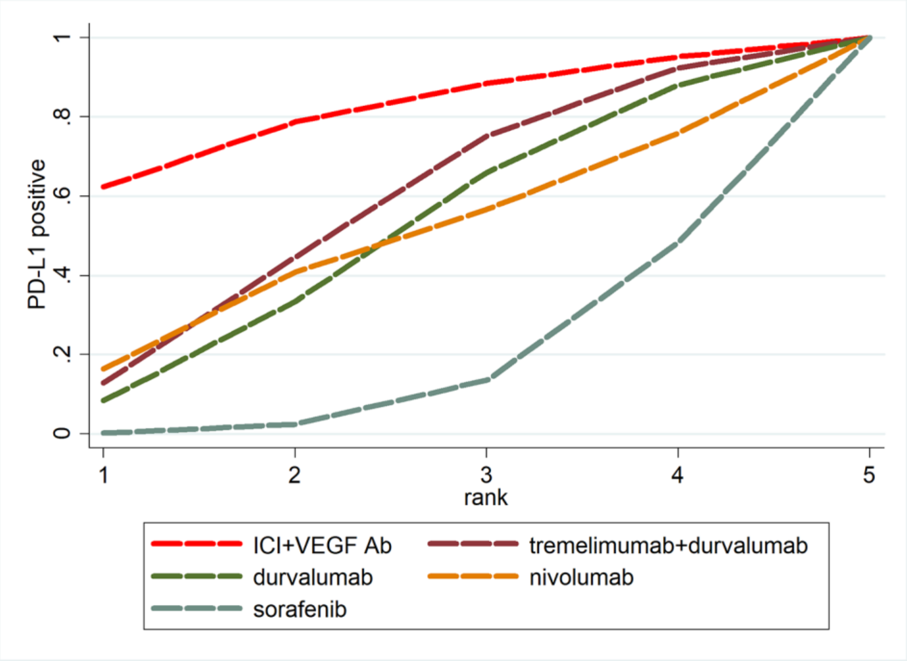

I. PD-L1 negaive

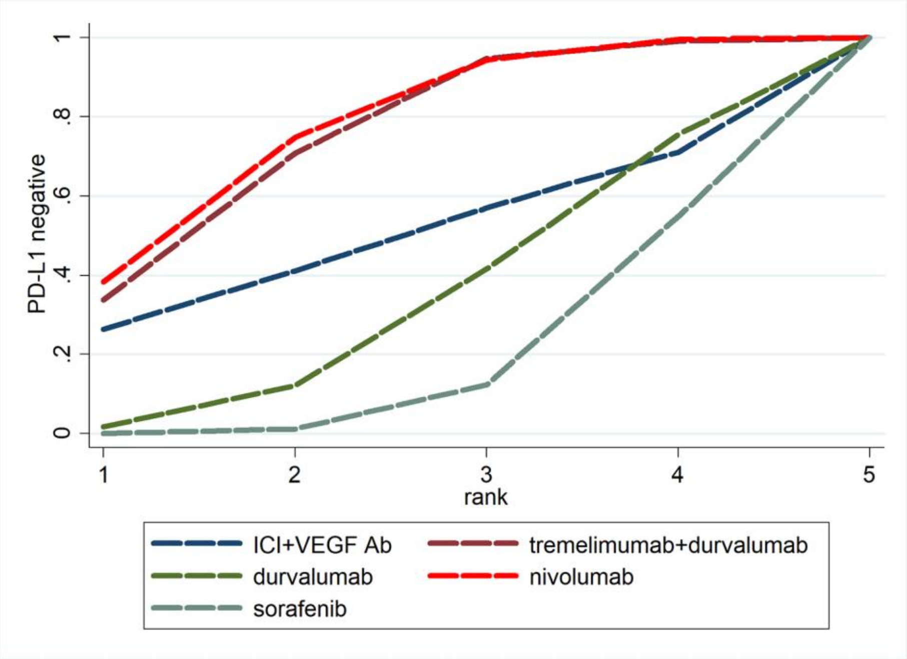

J. TRSAEs

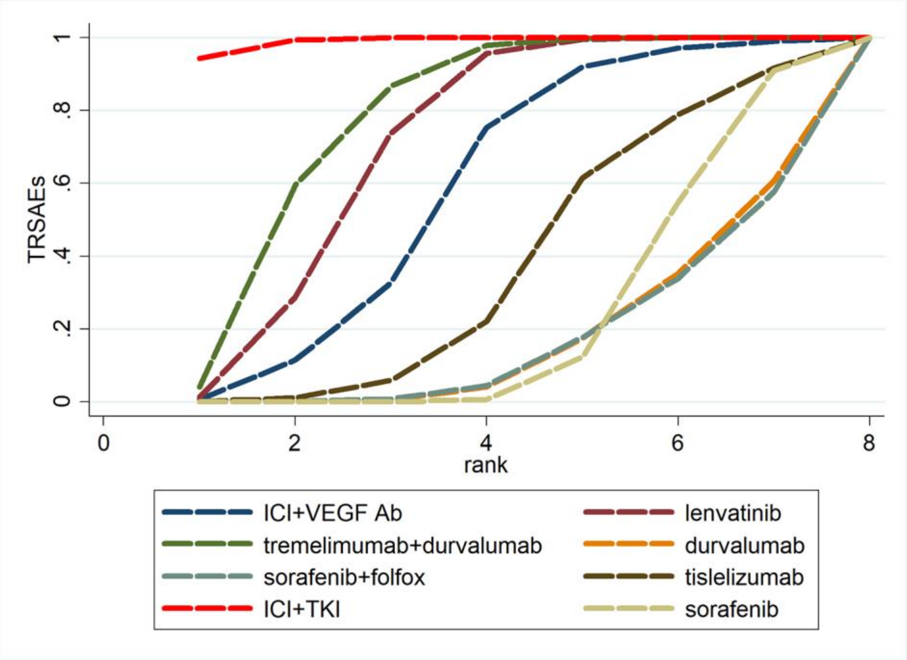

Abbreviations: ICI, immune checkpoint inhibitor; VEGF Ab, vascular endothelial growth factor antibody; TKIs, tyrosine kinase inhibitors; MVI, macrovascular invasion; EHS, extrahepatic metastasis; AEs, adverse events; TRSAEs, treatment related serious adverse events. Bayesian ranking profiles of comparable treatments on efficacy for patients with advanced hepatocellular carcinoma. The surface under the cumulative ranking curve (SUCRA) metric was used to

rank the effectiveness or safety of each treatment and identify the best treatment. A higher SUCRA value indicates a higher probability of the treatment regimen being ranked first.

eFigure 9. Stratified analysis focusing specifically on the time factor

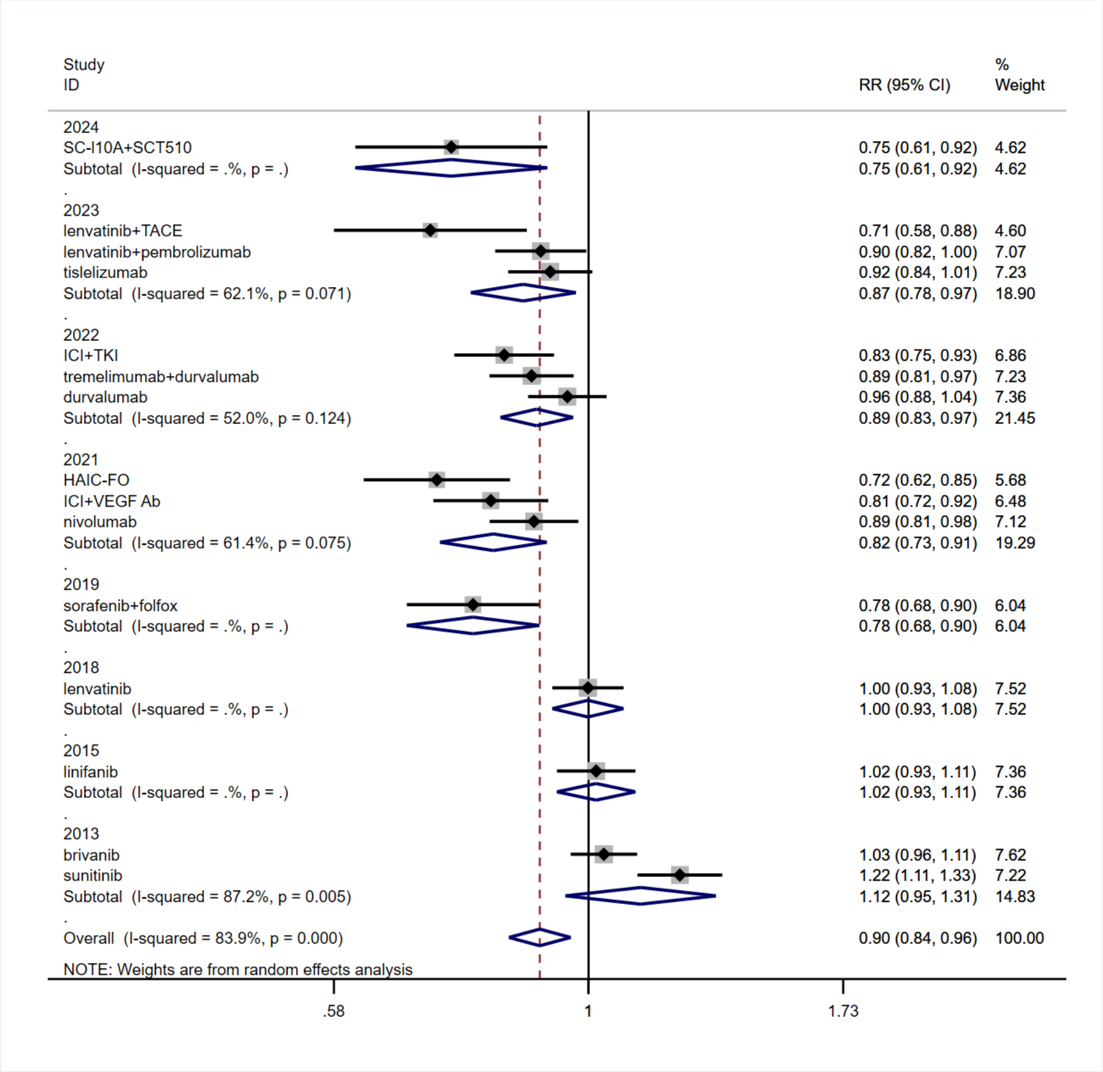

Abbreviations: ICI, immune checkpoint inhibitor; VEGF Ab, vascular endothelial growth factor antibody; TKIs, tyrosine kinase inhibitors; MVI, macrovascular invasion; EHS, extrahepatic metastasis; AEs, adverse events; TRSAEs, treatment related serious adverse events.
